# Comparison of the efficacy and comfort of high-flow nasal cannula with different initial flow settings in patients with acute hypoxemic respiratory failure: a systematic review and network meta-analysis

**DOI:** 10.1186/s40560-023-00667-2

**Published:** 2023-05-10

**Authors:** Yuewen He, Xuhui Zhuang, Hao Liu, Wuhua Ma

**Affiliations:** 1 Guangzhou University of Chinese Medicine, Guangzhou, Guangdong People’s Republic of China; 2 Department of Anesthesiology, The First Affiliated Hospital of Guangzhou University of Chinese Medicine, 12 Jichang Road, Guangzhou, Guangdong 510405 People’s Republic of China

**Keywords:** High-flow nasal cannula, Acute hypoxemic respiratory failure, Network meta-analysis

## Abstract

**Background:**

High-flow nasal cannula (HFNC) has been proven effective in improving patients with acute hypoxemic respiratory failure (AHRF), but a discussion of its use for initial flow settings still need to be provided. We aimed to compare the effectiveness and comfort evaluation of HFNC with different initial flow settings in patients with AHRF.

**Methods:**

Studies published by October 10, 2022, were searched exhaustively in PubMed, Embase, Web of Science, Cochrane Library (CENTRAL), and the China National Knowledge Infrastructure (CNKI) database. Network meta-analysis (NMA) was performed with STATA 17.0 and R software (version 4.2.1). A Bayesian framework was applied for this NMA. Comparisons of competing models based on the deviance information criterion (DIC) were used to select the best model for NMA. The primary outcome is the intubation at day 28. Secondary outcomes included short-term and long-term mortality, comfort score, length of ICU or hospital stay, and 24-h PaO_2_/FiO_2_.

**Results:**

This NMA included 23 randomized controlled trials (RCTs) with 5774 patients. With NIV as the control, the HFNC_high group was significantly associated with lower intubation rates (odds ratio [OR] 0.72 95% credible interval [CrI] 0.56 to 0.93; moderate quality evidence) and short-term mortality (OR 0.81 95% CrI 0.69 to 0.96; moderate quality evidence). Using HFNC_Moderate (Mod) group (mean difference [MD] − 1.98 95% CrI -3.98 to 0.01; very low quality evidence) as a comparator, the HFNC_Low group had a slight advantage in comfort scores but no statistically significant difference. Of all possible interventions, the HFNC_High group had the highest probability of being the best in reducing intubation rates (73.04%), short-term (82.74%) and long-term mortality (67.08%). While surface under the cumulative ranking curve value (SUCRA) indicated that the HFNC_Low group had the highest probability of being the best in terms of comfort scores.

**Conclusions:**

The high initial flow settings (50–60 L/min) performed better in decreasing the occurrence of intubation and mortality, albeit with poor comfort scores. Treatment of HFNC for AHRF patients ought to be initiated from moderate flow rates (30–40 L/min), and individualized flow settings can make HFNC more sensible in clinical practice.

**Supplementary Information:**

The online version contains supplementary material available at 10.1186/s40560-023-00667-2.

## Introduction

AHRF is an urgent and life-threatening condition caused by various etiologies [1]. It is defined as a respiratory rate (RR) greater than 25 breaths/min and a PaO_2_/FIO_2_ ratio less than or equal to 300 mmHg, with no increase in PaCO_2_ [2, 3]. The clinical consequences of AHRF are comparable to that of acute respiratory distress syndrome (ARDS), which usually requires endotracheal intubation and invasive mechanical ventilation (IMV) to maintain normal oxygenation [4, 5]. Although IMV is a safe and efficient means of oxygenation in the short term, there is conclusive evidence that its use for more than 36 h can cause an inflammatory lung response coupled with ventilator-induced lung injury, which can exacerbate patients’ mortality [6, 7]. Thus, reducing unnecessary IMV and finding alternative NIV strategies to bridge the gap with IMV remains the main goal in treating patients with AHRF.

Various non-invasive oxygenation strategies have recently been developed to support oxygenation, with HFNC being a relatively new approach to oxygen treatment. HFNC can reduce the risk of ventilator-induced lung injury and mortality by delivering 60–70 L/min of warmed and humidified high-flow gas into the patient's nasal cavity via a nasal cannula, which can better match the AHRF patient's inspiratory needs and permit a fraction of inspired oxygen (FiO_2_) of up to 1.0 [8, 9]. In addition, it can provide low-level positive end-expiratory pressure (PEEP) in the upper airways, facilitating alveolar recruitment. Therefore, clinical practice guidelines strongly recommend using HFNC over NIV in the AHRF population [1, 10]. Despite the large number of RCTs studying HFNC in adult patients with AHRF, there is still a lack of current consensus on the criteria for initial flow settings [11, 12]. The flow setting of the HFNC is essential given that the physiological effects of the HFNC are flow related. As the flow rate changes, the patient's RR, inspiratory effort, dynamic lung compliance, and treatment comfort will change correspondingly [13]. Therefore, finding the optimal initial flow rate plays a pivotal role in the treatment of HFNC. A physiological study found that individualized the flow rate of HFNC significantly reduced inspiratory work and improved lung oxygenation [14]. Researchers hold divergent opinions, and relevant information is conflicting. Initial flow settings vary even in populations with the exact etiology [15, 16], which invariably increases the heterogeneity of studies [17].

To our knowledge, no systematic reviews and meta-analyses have been performed to compare different initial flow settings of HFNC in patients with AHRF. While optimal oxygen flow management is an important aspect of using HFNC. Therefore, there is a need for a methodologically rigorous and clinically useful study that will contribute to the management of HFNC. Our systematic review and network meta-analysis aimed to set up groups with different initial flow settings to assess the impact of HFNC initial flow rate settings on the efficacy and comfort of patients with AHRF.

## Methods

### Study protocol

This systematic review was designed according to the Preferred Reporting Items for Systematic Review and Meta-Analyses extension statement for reviews incorporating network meta-analyses. The PRISMA NMA checklist is available in Additional file 1: Table S1. The PROSPERO registration number is CRD42022343981.

### Search strategy

**Fig. 1:**
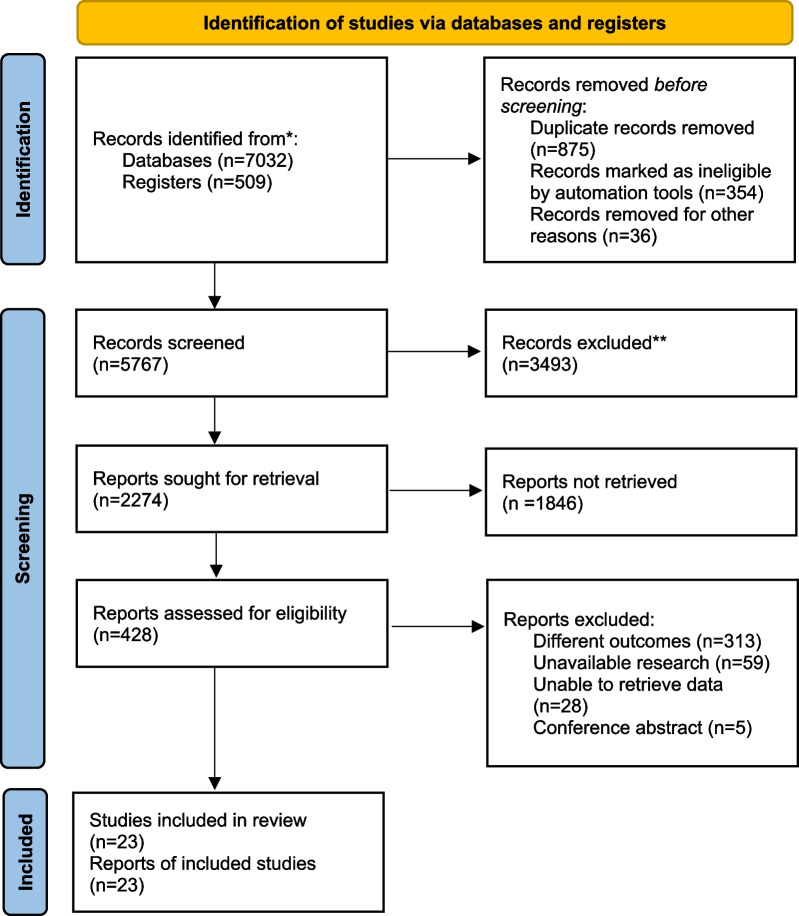
PRISMA flow diagram of the search strategy and included studies

The search process was shown in PRISMA_2020_flow_diagram (Fig. 1). Two researchers (Y.W.H. and X.H.Z.) exhaustively searched studies published from inception to October 10, 2022, without language restriction in PubMed, Embase, Web of Science, Cochrane Library (CENTRAL) and China National Knowledge Infrastructure (CNKI) database. The search formula was co-designed by two independent researchers. W.H.M. was responsible for resolving all disputes during the process. Synonym queries and similar terms of critical meta-analysis determined the search terms for this NMA. Based on different databases, we would appropriately change the retrieval strategy, such as Mesh word and Publication Type and other limitations. In addition, we will use different search formulas for different databases to avoid omissions (details in Additional file 1: Table S2).

### Study selection

The retrieved articles were managed by two researchers (Y.W.H. and X.H.Z.) using EndNote X9 (Thomson Reuters, NY, USA), respectively. The process was as follows: we excluded all duplicates and incomplete studies at first. And then, we reviewed the titles, keywords, and abstracts and graded them as “low correlation”, “moderate correlation” and “high correlation” based on inclusion criteria. Investigators excluded all “low correlation” studies and examined the full text of remaining studies defined as “moderate correlation”, as well as all studies with “high correlation”. Finally, two researchers identified the included literature based on the full text. When the results of the two researchers diverged, the opinion of a researcher (W.H.M.) was used to reach a consensus. Figure 1 includes a screening process to illustrate the number of excluded studies at each stage.

### Eligibility criteria

For the inclusion of this NMA, studies had to meet the following criteria: study type including completed and published RCTs with no language restriction; participants containing adult patients aged > 18 years with AHRF; interventions including studies requiring continuous HFNC or NIV ventilation treatment due to AHRF. There is no restriction on the type of NIV and the cause of ARHF, which is to fully evaluate the effectiveness of different initial flow settings of HFNC for AHRF. Besides, the exclusion criteria were as follows: study type: case report, review, animal experiment, consensus, protocol, NRCTs, and unpublished RCTs; participants: patients aged < 18 years or aged > 18 years with hypercapnic respiratory failure; outcomes: physiological indicators.

### Data extraction

Three investigators (Y.W.H., X.H.Z. and H.L.) were responsible for data extraction independently, and W.H.M. resolved all the disputes. If the result is represented graphically, we use WebPlotDigitizer (WebPlotDigitizer. Version: 4.4. Ankit Rohatgi. Pacifica, California, USA. November 2020) to measure and extract the data. During the data extraction phase, we converted the median (inter-quartile range, IQR) and median (range) to mean (standard deviation, SD) according to the method proposed by Wan et al. [18]. Table 1 summarizes the characteristics of the patients. The interventions of included studies are demonstrated in Table 2. We extracted the following data based on the characteristics of the included studies: Author, National, Year of publication, Type of hospital, Age, Gender, BMI, and Causes of AHRF in Table 1. Moreover, we extracted the Sources, Groups, Oxygen therapy apparatus, Oxygenation strategy and Initial flow settings in Table 2. Y.W.H. and H.L. extracted and summarized the research data in Excel 2019, and W.H.M. was responsible for confirming the accuracy of the research data.

**Table 1:** The patients’ characteristics of the included studies (*n* = 23)

Author, nationality	Type of hospital	Causes of AHRF	Groups: *n*	Ages (years)	Gender (male/female)	*P*/*F* ratio	PaCO2 (mmHg)	Type of patients
Alptekinoğlu et al. 2021 Turkey	Academic hospital	Pneumonia, extra-pulmonary sepsis, pleural effusion, non-infections pulmonary disease, CHF, ARDS and other	HFNC: 51	Mean (SD): 57(15)	33/18	Mean (SD): 256(19)	Mean (SD): 30(5)	Immunocompromised patients
			NIV: 49	Mean (SD): 58(12)	33/16	Mean (SD): 270(23)	Mean (SD): 32(5)	
Andino et al. 2020 Spanish	Academic hospital	Community/ hospital-acquired pneumonia, adult respiratory distress, pulmonary embolism and other diagnoses	HFNC: 24	Mean (SD): 58(19)	13/11	Mean (SD): 96(29)	Mean (SD): 35(6)	NR
			NIV: 22	Mean (SD): 61(11)	13/9	Mean (SD): 95(37)	Mean (SD): 34(7)	
Azoulay et al. 2018 France	24 university-affiliated, 8 non–university-affiliated hospitals	Chronic respiratory diseases, heart failure, liver/kidney diseases	HFNC: 388	Median (IQR): 64(55–70)	270/118	Median (IQR): 136(96–187)	NR	Immunocompromised patients
			NIV: 388	Median (IQR): 63(56–70)	147/141	Median (IQR): 128(92–164)	NR	
Bell et al. 2015 Australia	Tertiary referral hospitals	NR	HFNC: 48	Mean (SD): 72.9(14)	20/28	NR	NR	NR
			NIV: 52	Mean (SD): 72.9(15.1)	24/28	NR	NR	
Coudroy et al. 2022 France	Academic hospital	NR	HFNC: 154	Mean (SD): 62(13)	95/59	Mean (SD): 148(56)	Mean (SD): 34(6)	Immunocompromised patients
			NIV: 145	Mean (SD): 65(12)	97/48	Mean (SD): 147(57)	Mean (SD): 35(6)	
Feng et al. 2020 China	Academic hospital	Community acquired pneumonia, Hospital acquired pneumonia, Extrapulmonary sepsis, other	HFNC:40	Mean (SD): 60(9)	27/13	Mean (SD): 154(61)	Mean (SD): 32(5)	NR
			SO: 39	Mean (SD): 55(13)	22/17	Mean (SD): 142(67)	Mean (SD): 36(4)	
			NIV: 37	Mean (SD): 57(11)	22/15	Mean (SD): 148(62)	Mean (SD): 39(5)	
Frat et al. 2015 France	Academic hospital	Community acquired pneumonia, Hospital acquired pneumonia, Extrapulmonary sepsis, aspiration or drowning, Pneumonia-related to immunosuppression, Other	HFNC: 106	Mean (SD): 61(16)	75/31	Mean (SD): 157(89)	Mean (SD): 36(6)	NR
			SO: 94	Mean (SD): 59(17)	63/31	Mean (SD): 161(73)	Mean (SD): 35(5)	
			NIV: 110	Mean (SD): 61(17)	74/36	Mean (SD): 149(72)	Mean (SD): 34(5)	
Frat et al. 2022 France	34 ICUs in France	COVID-19	HFNC: 357	Mean (SD): 61(12)	250/107	Mean (SD): 128(31)	Mean (SD): 35(5)	COVID-19 patients
			NIV: 354	Mean (SD): 61(12)	247/107	Mean (SD): 132(31)	Mean (SD): 35(4)	
Grieco et al. 2021 Italy	Academic hospital	COVID-19	HFNC: 55	Median (IQR): 63(55–69)	46/9	Median (IQR): 102(80–124)	Median (IQR): 34(32–37)	COVID-19 patients
			NIV: 54	Median (IQR): 66(57–72)	42/12	Median (IQR): 105(83–125)	Median (IQR): 34(31–37)	
Jones et al. 2016 New Zealand	A tertiary academic inner-city hospital	NR	HFNC: 165	Mean (SD): 74.6(15.6)	73/92	NR	NR	NR
			NIV: 138	Mean (SD): 72.2(16.8)	71/67	NR	NR	
Lemiale et al. 2015 France	Academic hospital	Sepsis, cardiogenic pulmonary edema, noninfectious pulmonary disease, lung involvement by the underlying disease, large pleural effusion, Pneumocystis pneumonia, miscellaneous, no diagnosis	HFNC: 52	Median (IQR): 59.3(43–70)	38/14	Median (IQR): 128(48–178)	NR	Immunocompromised patients
			NIV: 48	Median (IQR): 64.5(53.25–72)	32/16	Median (IQR): 100 (40–156)	NR	
Lemiale et al. 2016 France	Academic hospital	Infection, Cardiogenic edema, Opportunistic infection, fungal infection, others	HFNC: 127	Median (IQR): 64 (53–72)	85/42	NR	NR	Immunocompromised patients
			NIV: 226	Median (IQR): 63 (52–70)	63/163	NR	NR	
Liu et al. 2018 China	NR	NR	HFNC: 40	Mean (SD): 50.85(7.53)	22/18	NR	NR	Immunocompromised patients
			NIV: 40	Mean (SD): 52. 31(6.78)	24/16	NR	NR	
Nair et al. 2021 India	ICU in All India Institute of Medical Sciences	COVID-19	HFNC: 55	Median (IQR): 57(48–65)	44/11	Median (IQR):105.0 (92.0–139.3)	Median (IQR):34 (26.3–38.5)	COVID-19 patients
			NIV: 54	Median (IQR): 57.5(47–64)	35/19	Median (IQR):111.2 (89.8–145.0)	Median (IQR):32 (26.0–43.3)	
OspinaTascón et al. 2021 Colombia	NR	COVID-19	HFNC: 99	Median (IQR): 60(50–69)	71/28	Median (IQR): 104(85–132)	Median (IQR): 32(30–35)	COVID-19 patients
			NIV: 100	Median (IQR): 59(49–67)	63/37	Median (IQR): 105(85–141)	Median (IQR): 32(30–36)	
Perkins et al. 2022 UK	48 acute care hospitals in the UK and Jersey	COVID-19	HFNC: 418	Mean (SD): 57.6(13.0)	272/146	Median (IQR): 115.0 (80.9–168.4)	Median (IQR): 33.0 (30.0–36.0)	COVID-19 patients
			NIV: 380	Mean (SD): 56.7(12.5)	260/120	Median (IQR): 112.5 (80.0–161.3)	Median (IQR): 33.0 (30.0–36.8)	
			COT: 475	Mean (SD): 57.6(12.7)	312/163	Median (IQR): 113.8 (84.8–150.9)	Median (IQR): 33.8 (30.8–36.8)	
Qiao et al. 2021 China	University-affiliated hospital	AIS	HFNC: 50	Mean (SD): 68.7(10.2)	30/20	Mean (SD): 223.3(17.8)	NR	AIS patients
			NIV: 48	Mean (SD): 70.4(12.4)	30/18	Mean (SD): 217.3(18.6)	NR	
Rittayamai et al. 2015 Thailand	Academic hospital	NR	HFNC: 20	Mean (SD): 65.6(14.4)	9/11	NR	NR	NR
			NIV: 20	Mean (SD): 63.6(15.7)	6/14	NR	NR	
Stephan et al. 2015 France	NR	Cardiothoracic surgery	HFNC: 414	Median (IQR): 63.8 (62.5–65.2)	273/141	Median (IQR): 196 (187–204)	Median (IQR): 38.7 (38.1–39.4)	Cardiothoracic surgery patients
			NIV: 416	Median (IQR): 63.9 (62.6–65.2)	278/138	Median (IQR): 203 (195–212)	Median (IQR): 39.1 (38.4–39.8)	
Vourc'h et al. 2019 France	Academic hospital	Atelectasis, Hemothorax, Acute colonic pseudo-obstruction, cardiogenic pulmonary edema, pneumonia, Pericardial effusion	HFNC: 47	Mean (SD): 65.8(10.1)	41/6	Mean (SD): 147.7(30.7)	Mean (SD): 40.5(3.8)	Cardiothoracic surgery patients
			NIV: 43	Mean (SD): 67.6(9.4)	36/7	Mean (SD): 131.5(27.7)	Mean (SD): 39.8(4.5)	
Wang et al. 2018 China	NR	NR	HFNC: 28	Mean (SD): 63.8(12.5)	16/12	NR	Mean (SD): 29.1(2.8)	NR
			NIV: 28	Mean (SD): 62.4(13.3)	17/11	NR	Mean (SD): 30.2(3.6)	
Zhao et al. 2019 China	University-affiliated hospitals	NR	HFNC: 80	Mean (SD): 56.32(5.23)	42/38	Mean (SD): 137.5(34.2)	Mean (SD): 37.36(3.74)	NR
			NIV: 80	Mean (SD): 55.86(6.35)	44/36	Mean (SD): 142.9(32.4)	Mean (SD): 38.09(4.05)	
Zeng et al. 2019 China	NR	NR	HFNC: 62	Mean (SD): 56.94(9.46)	32/30	Mean (SD): 164.4(21.5)	NR	NR
			NIV: 62	Mean (SD): 57.23(9.73)	35/27	Mean (SD): 163.9(21.8)	NR	

*HFNC* high-flow nasal cannula, *NIV* non-invasive ventilation, *ARDS* acute respiratory distress syndrome, *CHF* congestive heart failure, *COPD* chronic obstructive pulmonary disease, *COVID-19* the novel coronavirus disease 2019, *AIS* Acute ischemic stroke, *PaO*_*2*_*/FiO*_*2*_* ratio* ratio of arterial oxygen partial pressure to fraction of inspired oxygen, *PaCO*_*2*_ partial pressure of arterial carbon dioxide, *RR* respiratory rate, *SD* standard deviation, *IQR* inter-quartile range, *NR* not recorded

**Table 2:** The intervention’s characteristics of included studies (*n* = 23)

Authors, nationality	Definition of AHRF	Groups	Initial flow settings	Oxygenation strategy	Oxygen therapy apparatus
Alptekinoğlu et al. 2021 Turkey	PaO_2_/FiO_2_ ratio < 300 mmHg or SpO_2_ < 92% on room air, PaCO_2_ ≤ 45 mmHg, RR > 22 breaths/min, or labored breathing with respiratory distress	HFNC	30 L/min	Temperature: NR Maximal flow rate: 50 L/min FiO_2_: 100% SpO_2_: ≥ 94% Duration of treatment: NR	NR
		NIV	6 L/min	Temperature: NR Maximal flow rate: NR FiO_2_: 100% SpO_2_: ≥ 94% Duration of treatment: NR	Nasal prongs or facial oxygen masks without reservoir bag
Andino et al. 2020 Spanish	PaO_2_/FiO_2_ ratio ≤ 200 mmHg or SpO_2_/FiO_2_ ratio ≤ 160 mmHg and RR > 30 breaths/ min for at least 30 min	HFNC	20 L/min	Temperature: 34–37 °C Maximal flow rate: 50 L/min FiO_2_: 60%-100% SpO_2_: ≥ 93% Duration of treatment: NR	Optiflow®, Fisher & Paykel, Maidenhead, UK
		NIV	15 L/min	Temperature: NR Maximal flow rate: 30 L/min FiO_2_: 60%-100% SpO_2_: ≥ 93% Duration of treatment: NR	Venturi mask
Azoulay et al. 2018 France	AHRF with PaO_2_ < 60 mm Hg or SpO_2_ < 90% on room air, or tachypnea > 30/min or labored breathing or respiratory distress; FiO_2_ ≥ 6 L/min	HFNC	50 L/min	Temperature: NR Maximal flow rate: 60 L/min FiO_2_: 100% SpO_2_: ≥ 95% Duration of treatment: NR	NR
		NIV	NR	Temperature: NR Maximal flow rate: 15 L/min FiO_2_: 100% SpO_2_: ≥ 95% Duration of treatment: NR	Any device or combination of devices used for standard care (nasal prongs or mask with or without a reservoir bag and with or without a Venturi system)
Bell et al. 2015 Australia	RR > 25 breaths/min and SpO_2_ < 93%	HFNC	50 L/min	Temperature: NR Maximal flow rate: NR FiO_2_: 30% SpO_2_: NR Duration of treatment: ≥ 2 h	AIRVO2, Optiflow, Fisher & Paykel, Auckland, New Zealand
		NIV	NR	Temperature: NR Maximal flow rate: 60 L/min FiO_2_: 100% SpO_2_: NR Duration of treatment: ≥ 2 h	Standard nasal prongs or face mask (Hudson, venturi system or non- rebreather)
Coudroy et al. 2022 France	RR ≥ 25 breaths /min, and PaO_2_/FiO_2_ ratio ≤ 300 mm Hg	HFNC	60L/min	Temperature: NR Maximal flow rate: NR FiO_2_:NR SpO_2_: ≥ 92% Duration of treatment: NR	Heated humidifier (MR 850, Fisher & Paykel Healthcare, Auckland, New Zealand)
		NIV	VT ≤ 8 ml/kg PEEP > 8 cmH_2_O	Temperature: NR Maximal flow rate: NR FiO_2_:NR SpO_2_: ≥ 92% Duration of treatment: NR	ICU ventilator after activation of non-invasive mode or non-invasive bilevel ventilator
Feng et al. 2020 China	RR ≥ 25 breaths/min, PaO_2_/FiO_2_ ratio ≤ 300 mmHg, and PaCO_2_ ≤ 45 mmHg	HFNC	50 L/min	Temperature: NR Maximal flow rate: NR FiO_2_: 100% SpO_2_: ≥ 93% Duration of treatment: ≥ 2d	Fisher and Paykel Healthcare
		SO	> 10 L/min	Temperature: NR Maximal flow rate: NR FiO_2_: NR SpO_2_: ≥ 93% Duration of treatment: ≥ 2d	Mask
		NIV	VT = 7–10 ml/kg, PEEP = 2–10 cmH_2_O	Temperature: NR Maximal flow rate: NR FiO_2_: NR SpO_2_: ≥ 93% Duration of treatment: ≥ 2d	Respironics BiPAP non-invasive ventilator
Frat et al. 2015 France	RR ≥ 25 breaths/min, PaO_2_/FiO_2_ ratio ≤ 300 mmHg while breathing oxygen ≥ 10 L/min for at least 15 min, PaCO_2_ ≤ 45 mmHg	HFNC	50 L/min	Temperature: NR Maximal flow rate: NR FiO_2_: 1.0 SpO_2_: ≥ 92% Duration of treatment: ≥ 2d	Heated humidifier (MR850, Fisher and Paykel Healthcare)
		SO	≥ 10 L/min	Temperature: NR Maximal flow rate: NR FiO_2_: NR SpO_2_: ≥ 92% Duration of treatment: NR	Nonrebreather face mask
		NIV	VT = 7–10 ml/kg, PEEP = 2–10 cmH_2_O	Temperature: NR Maximal flow rate: NR FiO_2_: NR SpO_2_: ≥ 92% Duration of treatment: ≥ 2d	Face mask (Fisher and Paykel Health- care)
Frat et al. 2022 France	PaO_2_/FiO_2_ ratio ≤ 200 mmHg while breathing oxygen at a flow rate of 10 L/min or more for at least 15 min	HFNC	50 L/min	Temperature: NR Maximal flow rate: NR FiO_2_: NR SpO_2_: 92–96% Duration of treatment: ≥ 48 h	Optiflow or Airvo-2, Fisher & Paykel Healthcare; or an ICU ventilator with a high-flow oxygen therapy option
		NIV	10L/min	Temperature: NR Maximal flow rate: NR FiO_2_: NR SpO_2_: 92%—96% Duration of treatment: ≥ 48 h	Nonrebreathing mask
Grieco et al. 2021 Italy	PaO_2_/FiO_2_ ratio ≤ 200 mmHg, PaCO_2_ ≤ 45 mmHg	HFNC	60 L/min	Temperature: 34 °C or 37 °C Maximal flow rate: 60 L/min FiO_2_: NR SpO_2_: 92%—98% Duration of treatment: ≥ 48 h	Fisher and Paykel Healthcare, New Zealand
		NIV	NR	Temperature: NR Maximal flow rate: NR FiO_2_: NR SpO_2_: 92%—98% Duration of treatment: 48 h	Helmet interface (Dimar, Italy, or Starmed-Intersurgical, UK)
Jones et al. 2016 New Zealand	SpO_2_ < 92% on air, RR ≥ 22 breaths/min	HFNC	40 L/min	Temperature: 37 °C Maximal flow rate: NR FiO_2_: 28% SpO_2_: 93% Duration of treatment: NR	Optiflow nasal interface connected to the PT101AX (Airvo1) or PT101AZ (Airvo2) humidifier (Fisher & Paykel Healthcare, Auckland, New Zealand)
		NIV	1–15 L/min	Temperature: NR Maximal flow rate: 15L/min FiO_2_: NR SpO_2_: 93% Duration of treatment: NR	HudNIVn mask, Venturi device, or standard nasal prongs
Lemiale et al. 2015 France	FiO_2_ > 6 L/min to maintain SpO_2_ > 95% or symptoms of respiratory distress (tachypnea > 30/min, intercostal recession, labored breathing, and/or dyspnea at rest)	HFNC	40–50 L/min	Temperature: NR Maximal flow rate: 50 L/min FiO_2_: 100% SpO_2_: ≥ 95% Duration of treatment: ≥ 2 h	Heated humidified circuit
		NIV	15 L/min	Temperature: NR Maximal flow rate: NR FiO_2_: 60% SpO_2_: ≥ 95% Duration of treatment: ≥ 2 h	Venturi mask
Lemiale et al. 2016 France	FiO_2_ > 6 L/min to maintain SpO_2_ > 95% or symptoms of respiratory distress (tachypnea > 30/min, intercostal recession, labored breathing, and/or dyspnea at rest)	HFNC	Median (Range): 40 (15–50) L/min	Temperature: NR Maximal flow rate: 50 L/min FiO_2_: NR SpO_2_: 92% Duration of treatment: NR	NR
		NIV	Median (Range): 5 (4–9) L/min	Temperature: NR Maximal flow rate: 9 L/min FiO_2_: NR SpO_2_: NR Duration of treatment: NR	NR
Liu et al. 2018 China	RR ≥ 30 breaths/min, PaO_2_/FiO_2_ ratio < 200 mmHg	HFNC	45 L/min	Temperature: 37 °C Maximal flow rate: NR FiO_2_: 60%-80% SpO_2_: NR Duration of treatment: NR	Fisher & Paykel's high-flow transnasal oxygenation devices and nasal plug catheters
		NIV	NR	Temperature: NR Maximal flow rate: NR FiO_2_: 60%-80% SpO_2_: NR Duration of treatment: NR	Phillip Non-invasive Ventilator V60
Nair et al. 2021 India	RR > 24 breaths/min and/or SpO_2_ < 94%	HFNC	50 L/min	Temperature: NR Maximal flow rate: 60 L/min FiO_2_: 1.0 SpO_2_: ≥ 94% Duration of treatment: NR	Large-bore binasal prongs with a high-flow heated humidifier device (Optiflow, Fisher & Paykel Healthcare, Auckland, New Zealand)
		NIV	VT = 7–10 mL/kg PEEP = 5–10 cmH_2_O	Temperature: NR Maximal flow rate: NR FiO_2_: 0.5—1.0 SpO_2_: ≥ 94% Duration of treatment: NR	Mask/helmet
OspinaTascón et al. 2021 Colombia	PaO_2_/FiO_2_ ratio ≤ 200 mmHg, and respiratory distress (use of accessory muscles and RR > 25/min)	HFNC	60 L/min	Temperature: NR Maximal flow rate: NR FiO_2_: 100% SpO_2_: ≥ 92% Duration of treatment: NR	Large-bore binasal prongs using heated and humidified gas
		NIV	NR	Temperature: NR Maximal flow rate: NR FiO_2_: NR SpO_2_: ≥ 92% Duration of treatment: NR	Nasal prongs, mask with or without oxy- gen reservoir, Venturi mask systems
Perkins et al. 2022 UK	SpO_2_ ≤ 94% when FiO_2_ ≥ 0.40	HFNC	Mean (95% CI): 52.4 (51.4–53.5) L/min	Temperature: NR Maximal flow rate: NR FiO_2_: NR SpO_2_: NR Duration of treatment: 3.7 (4.1) d	Heated humidified HFNC
		NIV	NR	Temperature: NR Maximal flow rate: NR FiO_2_: NR SpO_2_: NR Duration of treatment: 3.5 (4.6) d	Standard face mask or low-flow nasal cannula
Qiao et al. 2021 China	PaO_2_ < 60 mmHg and PaCO_2_ < 50 mmHg	HFNC	2–60 L/min	Temperature: 31–37 °C Maximal flow rate: 60 L/min FiO_2_: 21%-100% SpO_2_: > 94% Duration of treatment: NR	Fisher & Paykel Respiratory Humidifier (AIRVO2, manufactured by Fishser & Paykel, New Zealand)
		NIV	NR	Temperature: NR Maximal flow rate: NR FiO_2_: NR SpO_2_: > 94% Duration of treatment: NR	Oxygen via nasal / face mask
Rittayamai et al. 2015 Thailand	RR > 24 breaths/min and SpO_2_ < 94% on the room air	HFNC	35L/min	Temperature: 37 °C Maximal flow rate: 60L/min FiO_2_: NR SpO_2_: ≥ 94% Duration of treatment: NR	Optiflow, Fisher & Paykel Healthcare, Auckland, New Zealand
		NIV	3-10L/min	Temperature: 37 °C Maximal flow rate: NR FiO_2_: NR SpO_2_: ≥ 94% Duration of treatment: NR	Nasal cannula or non-rebreathing mask
Stephan et al. 2015 France	PaO_2_/FiO_2_ ratio < 300 mmHg, RR > 25 breaths/min for at least 2 h	HFNC	50 L/min	Temperature: 37 °C Maximal flow rate: NR FiO_2_: 50% SpO_2_: 92%—98% Duration of treatment: NR	Nasal cannula with Optiflow (Fisher and Paykel Healthcare)
		NIV	VT = 9 ml/kg PEEP = 4 cmH_2_O PS = 8 cmH_2_O	Temperature: NR Maximal flow rate: NR FiO_2_: 50% SpO_2_: 92%—98% Duration of treatment: NR	Full-face mask
Vourc'h et al. 2019 France	Severe hypoxemia defined as SpO_2_ < 96% with Venturi mask with FiO_2_ of 50%	HFNC	45 L/min	Temperature: 37 °C Maximal flow rate: NR FiO_2_: 100% SpO_2_: NR Duration of treatment: 48 h	(Optiflow, Fisher & Paykel Healthcare, Auckland, New Zealand)
		NIV	15 L/min	Temperature: NR Maximal flow rate: NR FiO_2_: 100% SpO_2_: NR Duration of treatment: 48 h	Hudson RCI non-rebreather mask with a reservoir bag
Wang et al. 2018 China	PaO_2_ < 60 mmHg, RR > 25 breaths/min, PaO_2_/FiO_2_ ratio ≤ 200 mmHg, PaCO_2_ < 45 mmHg	HFNC	50 L/min	Temperature: NR Maximal flow rate: NR FiO_2_: 100% SpO_2_: ≥ 92% Duration of treatment: NR	High-flow ventilator (Fisher & Paykel New Zealand, Airvo2)
		NIV	5–10 L/min	Temperature: NR Maximal flow rate: 10L/min FiO_2_: NR SpO_2_: ≥ 92% Duration of treatment: NR	Nose and mouth mask, non-invasive ventilator
Zhao et al. 2019 China	PaO_2_ < 60 mmHg, RR > 25 breaths/min, PaO_2_/FiO_2_ ratio ≤ 200 mmHg, PaCO_2_ < 45 mmHg	HFNC	50 L/min	Temperature: NR Maximal flow rate: NR FiO_2_: 100% SpO_2_: ≥ 92% Duration of treatment: 48 h	Airvo2 Heated Humidified High Flow Dual Chamber Nasal Oxygenator (Fisher & Paykel, New Zealand)
		NIV	5–10 L/min	Temperature: NR Maximal flow rate: NR FiO_2_: NR SpO_2_: ≥ 92% Duration of treatment: 48 h	Non-invasive ventilator V60 with oral and nasal mask (Philips Respironics, The Netherlands)
Zeng et al. 2019 China	RR ≥ 25 breaths/min, PaO_2_/FiO_2_ ratio ≤ 200 mmHg	HFNC	50 L/min	Temperature: NR Maximal flow rate: NR FiO_2_: 100% SpO_2_: ≥ 92% Duration of treatment: ≥ 48 h	Fisher and paykel healthcare
		NIV	VT = 6–10 mL/kg PEEP > 2 cmH_2_O	Temperature: NR Maximal flow rate: NR FiO_2_: NR SpO_2_: ≥ 92% Duration of treatment: ≥ 48 h	PHILIPS V60 ventilator

*HFNC* high-flow nasal cannula, *NIV* non-invasive ventilation, *ARDS* acute respiratory distress syndrome, *PaO*_*2*_*/FiO*_*2*_* ratio* ratio of arterial oxygen partial pressure to fractional inspired oxygen, *PaCO*_*2*_ partial pressure of arterial carbon dioxide, *SpO*_*2*_ oxygen saturation by pulse oximetry, *RR* respiratory rate, *VT* tidal volume, *PEEP* positive end-expiratory pressure, *CI* confidence interval, *NR* not recorded

### Risk of bias assessment

Two investigators independently assessed the risk of bias for each trial using Review Manager 5.4 (RevMan, The Cochrane Collaboration, Oxford, United Kingdom) according to the criteria outlined in the Cochrane Handbook for Systematic Reviews of Interventions [19]. Based on the Cochrane Collaboration’s tool, RCT was defined as high risk, low risk, and unclear. The risk of bias summary is shown in Additional file 1: Fig. S1. Furthermore, we chose meta packages of R (version 4.2.1) to generate funnel plots to assess publication bias. Evaluation methods include the plot of effect size centered at comparison-specific pooled effect and the Egger’s test to evaluate small sample effect. When researchers disagree on the biased analysis of the same study, another researcher (W.H.M.) will make the decision.

### Outcomes

The primary outcome is the intubation at day 28. The secondary outcomes included short-term mortality (within 30 days), long-term mortality (within 90 days), comfort scores, length of ICU and hospital stay, and 24-h PaO_2_/FiO_2_ ratio.

### Statistical analysis for pairwise meta-analysis

Two investigators (Y.W.H. and X.H.Z.) are responsible for the statistical methodology. Meta packages of R (version 4.2.1) were applied to perform the pairwise meta-analysis of direct evidence by using random-effects models or fixed-effects models (also called common effect models in meta packages of R 4.2.1). For the pairwise meta-analysis, heterogeneity between studies was estimated by the *I*-squared (*I*^2^) test and Cochran's *Q* test. According to the Cochrane Collaboration Handbook, when moderate or high heterogeneity (*I*^2^ > 50% and *P* < 0.05) was observed, a random-effects model was used; otherwise, a fixed-effects model was used.

### Statistical analysis for network meta-analysis

The HFNC was separated into three levels according to the initial flow settings and previous studies [20, 21]: (1) flow rate less than 35 L/min belongs to HFNC_Low; (2) flow rate between 35 and 50 L/min as HFNC_Mod; (3) the flow rate of more than 50L/min goes to HFNC_High. Stata 16.0 (StataCorp LLC, College Station, TX, USA) was used to generate network plots for different comparisons, visualizing the relationship between various interventions. The node size in the network plot represents the sample size of the group, and the edge width represents the number of studies.

For the NMA, the analysis was conducted in a Bayesian framework. The network estimates are obtained by the Markov chain Monte Carlo simulation method. For the analysis results of this study, two-tailed tests with *P* < 0.05 were defined as statistically significant. The metafor package (R 4.2.1) generated the NMA forest plot. Then, the deviance information criterion (DIC) and potential scale reduced factor (PSRF) were calculated. DIC is widely used in the selection of Bayesian models. In general, a smaller DIC indicates a better fit for the model [22]. As for the PSRF, closer to 1, means that the results have good convergence, and the consistency model can be considered robust (Additional file 1: Table S3). In addition, none of the NMA comparisons was a closed loop, so no inconsistency tests were performed.

Subsequently, we used BUGSnet packages of R (version 4.2.1) to calculate the surface under the cumulative ranking (SUCRA) to rank the interventions [23]. For the outcomes in this NMA, a larger value of SUCRA means a better effect. The SUCRA statistic ranges from 0 to 100%, and it indicates the likelihood that therapy will be ranked as the best therapy in the NMA [24]. Finally, meta-regression and subgroup analyses were performed for sources of heterogeneity.

### Certainty assessment of the evidence

Two independent investigators (Y.W.H. and H.L.) assessed the quality of the evidence by using the standard Grading of Recommendations Assessment, Development and Evaluation (GRADE) method. The NMA findings were evaluated comprehensively in terms of risk of bias, indirectness, inconsistency, imprecision, and publication bias according to the GRADE methodology [25]. Additionally, the GRADE published framework was used to guide the development of summary of findings (SoF) tables to report comparative results for the NMA [26].

## Results

### Literature search findings

We searched five databases with a total of 7541 studies (PubMed: 1213; Embase: 2267; Web of Science: 2766; Cochrane Library (CENTRAL): 878; CNKI: 417). We removed duplicate and ineligible studies, then excluded all studies defined as “low correlation”, and 428 RCTs were included. After co-screening the full text of 428 studies by two investigators, 23 studies were included in the NMA with 5774 patients. The search process is represented in PRISMA_2020_flow_diagram (Fig. 1).

### Study and patient characteristics

In total, 23 RCTs involving 5774 patients were included in this NMA. Table 1 summarizes the characteristics of the patients in the included studies. The ages, PaO_2_/FiO_2_ ratio and PaCO_2_ value were reported using mean (SD) or median (IQR). Notably, five studies [27–31] stated that they included patients with COVID-19. And five studies [15, 32–35] included immunocompromised patients with AHRF. The patients enrolled by Andino [36] et al. had the lowest PaO_2_/FiO_2_ ratio, with mean values less than 100 mmHg in both groups.

### Intervention characteristics

The researchers extracted the intervention characteristics of included studies (Table 2). Six studies [28, 30, 31, 35, 37, 38] did not report on the initial start flow of NIV. Two studies [39, 40] divided the included patients into three groups: HFNC, SO and NIV. While we selected only the HFNC and NIV groups for comparison during the analysis. Interestingly, Azoulay et al. [16] did not restrict the oxygen therapy apparatus of the NIV group, which meant that paramedics could use any oxygen device to maintain normal oxygenation. In addition, Lemiale et al. [33] did not report therapeutic devices for HFNC or NIV.

### Assessment of risk of bias and certainty of the evidence

The risk of bias assessments for 23 RCTs is shown in Additional file 1: Fig. S1. All the included studies performed random sequence generation. Three studies [34, 41, 42] with unclear performance in allocation concealment. Blinding is a crucial part of evaluating the quality of RCTs. However, only the study published by Frat et al. [27] in 2022 explicitly managed to blind participants and personnel. Therefore, the performance bias was defined as “high risk” for most studies. Funnel plots were generated to assess the publication bias of the studies (Additional file 1: Fig. S2). For the outcomes with less than ten included studies, the test for funnel plot asymmetry was skipped according to the recommendations [43]. Moreover, the results of Egger’s test indicated that only short-term mortality had a risk of publication bias (Additional file 1: Table S6; Fig. S2B).

Based on the GRADE methodology, we evaluated the certainty of the evidence obtained by the NMA (Additional file 1: Table S4). The certainty of all evidence was between moderate and very low. The comparisons between flow rates were indirect due to the lack of a relevant RCT comparing different initial flow settings of the HFNC. In light of their major concerns with imprecision and indirectness, these comparisons were deemed to have low or very low confidence. What’s more, the NMA had no closed loops and failed to perform the inconsistency tests. Consequently, all indirect evidence was downgraded in terms of inconsistency.

### Pairwise meta-analysis

In the first phase of data analysis, we performed a pairwise meta-analysis of the intubation rate at day 28 for the primary outcome (16 RCTs containing 3976 patients), which showed that HFNC was substantially superior to NIV (OR 0.72 95% CI 0.55 to 0.95; *P* = 0.02) (Additional file 1: Fig. S3A). As for secondary outcomes, we evaluated short-term mortality, long-term mortality, comfort scores, length of ICU stay, length of hospital stay, and 24-h PaO_2_/FiO_2_ ratio (Additional file 1: Fig.S3). A total of 14 studies (3905 patients) were included in the analysis of short-term mortality, in which HFNC (OR 0.83 95% CI 0.71 to 0.97; *P* = 0.017) was significantly effective in reducing short-term mortality compared with NIV (Additional file 1: Fig. S3B). Five studies with 429 participants were included in comparing comfort scores, with no significant difference found between HFNC and NIV (MD 0.16 95% CI − 0.96 to 1.27; *P* = 0.783) (Additional file 1: Fig. S3D). Direct comparisons of other outcomes were not statistically significant.

### Network meta-analysis

**Fig. 2:**
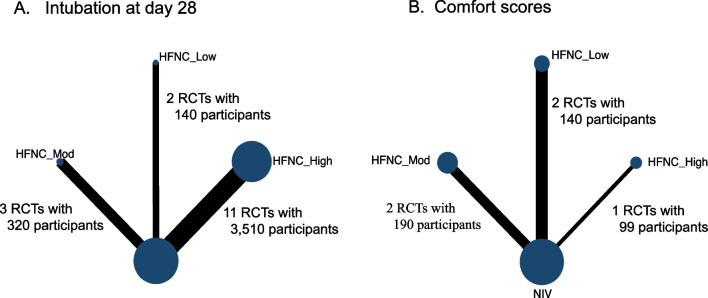
Network plot of intubation at day 28 (**A**) and comfort scores (**B**). The size of the node represents the number of participants who received the intervention. The thickness of lines connecting nodes represents the number of studies for that comparison

**Fig. 3:**
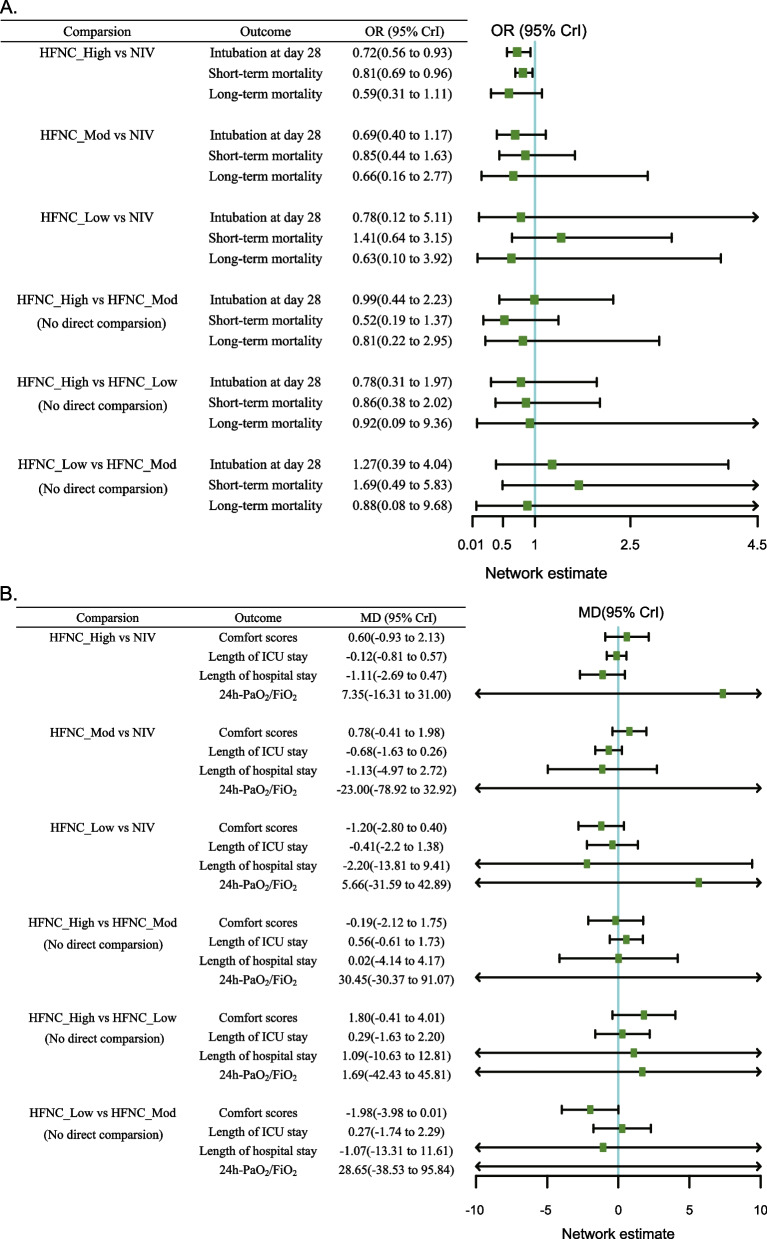
Forest plots of network meta-analysis. Intubation at day 28, short-term and long-term mortality were shown in **A**. Comfort scores, length of ICU stay and hospital stay, and 24-h PaO_2_/FiO_2_ were shown in **B**

Network plots for Intubation at day 28 (A) and Comfort scores (B) are presented in Fig. 2. Other outcomes of the network plots are shown in Additional file 1: Fig. S4. Direct comparisons occurred between HFNC and NIV, and comparisons between different initial flow rates of HFNC were only supported by indirect evidence. Since our comparisons of the initial flow settings of HFNC are indirect, node split analysis for the inconsistency test cannot be performed [44]. Pooled effect sizes from network estimates using the consistency model for the different comparisons are presented in Fig. 3. The random-effects model to generate the combined network effect values was selected depending on the DIC results (Additional file 1: Table S3).

**Fig. 4:**
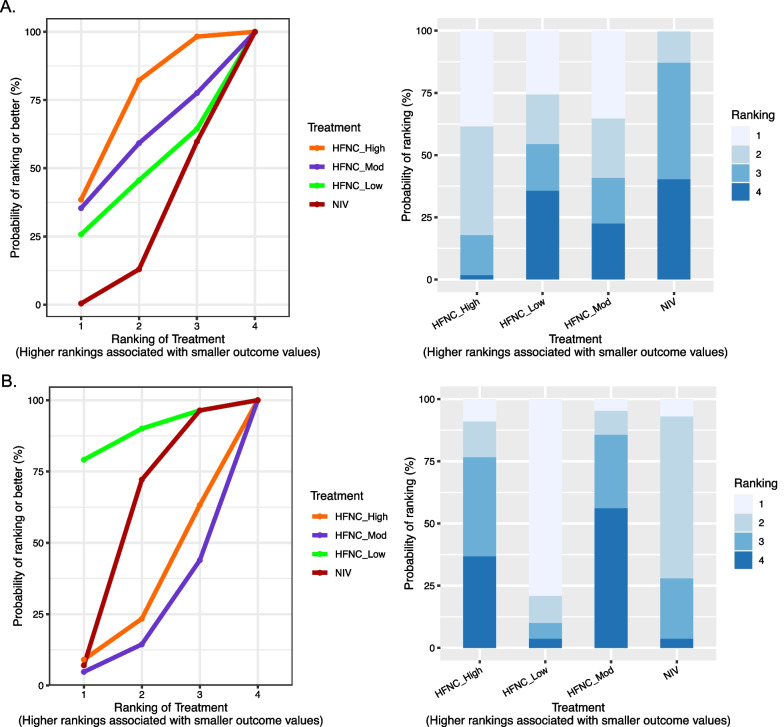
Line chart and bar chart of the surface under the cumulative ranking curve (SUCRA) values for intubation at day 28 (**A**) and comfort scores (**B**). The *x*-axis is the ranking of the initial flow rate setting and the y-axis is the cumulative probability of a particular ranking

Using the NIV as a comparator, only the HFNC_High group (OR 0.72, 95% CrI 0.56 to 0.93; moderate quality evidence) may modestly reduce the intubation rate at day 28 in patients with AHRF according to the network estimates. Although HFNC_Mod (OR 0.69 95% CrI 0.40 to 1.17; moderate quality evidence) was associated with a lower intubation rate, the 95% CI did not fully validate its effectiveness. Compared to NIV, the result of the HFNC_Low group (OR 0.78 95% CrI 0.12 to 5.11; low quality evidence) was not statistically significant. Compared with HFNC_Mod, neither HFNC_High (OR 0.99 95% CrI 0.44 to 2.23; very low quality evidence) nor HFNC_Low (OR 1.27 95% CrI 0.39 to 4.04; very low quality evidence) was associated with a statistically significant reduction in the risk of intubation. Moreover, the line chart and bar chart of SUCRA results are provided in Fig. 4 and Additional file 1: Fig.S4. The HFNC_High group (73.04%) has the highest SUCRA, followed by the HFNC_Mod group (57.52%), HFNC_Low group (44.53%) and NIV (22.90%) respectively (Fig. 4A; Additional file 1: Table S5).

Pooled effects from network estimates indicated that HFNC_High (OR 0.81 95% CrI 0.69 to 0.96; moderate quality evidence) was associated with lower short-term mortality than NIV. Using NIV as a reference, neither HFNC_Mod (OR 0.85 95% CrI 0.44 to 1.63; moderate quality evidence) nor HFNC_Low (OR 1.41 95% CrI 0.64 to 3.15; very low quality evidence) was statistically effective. In particular, the HFNC_Low group had only one RCT, yielding evidence with serious imprecision and risk of bias. No significant differences were found in the additional indirect comparisons. In SUCRA results, the HFNC_High group (82.74%) was the highest, while the HFNC_Low group (19.54%) had the worst performance (Additional file 1: Table S5; Fig. S3). As for long-term mortality, the results were not statistically significant, although the HFNC_high (OR 0.59 95% CrI 0.31 1.11; low quality evidence) group was associated with reduced mortality risk. The SUCRA results showed that the high flow group (67.08%) outperformed the moderate flow group (58.16%), the low flow group (51.66%) and the NIV group (23.11%) in terms of long-term mortality (Additional file 1: Table S5; Fig. S3).

Using the NIV as a reference, the initial flow setting in the HFNC_Low group (MD − 1.20 95% CrI − 2.80 to 0.04; low quality evidence) was the most comfortable for patients with AHRF, but the results were not statistically significant. Likewise, comfort scores were significantly better in the low flow group than in the moderate (MD − 1.98 95% CrI − 3.98 to 0.01; low quality evidence), albeit not statistically significant. Of all possible interventions, the HFNC_Low group (88.37%) had the highest probability of improving comfort scores, followed by NIV (57.94%), HFNC_High (32.07%), and HFNC_Mod (21.62%) (Fig. 4B; Additional file 1: Table S5). There were no significant differences in comparisons of 24-h PaO_2_/FiO_2_ ratio, length of ICU and hospital stay (Fig. 3B; Fig. S3). The SUCRA results suggested that HFNC_Mod has the highest probability of being the best treatment in terms of the length of ICU stay (78.29%) and 24-h PaO_2_/FiO_2_ (70.72%). In addition, as for the length of hospital stay, the probabilities being best was similar for the low (59.90%), moderate (55.21%), and high flow (60.28%) groups, with NIV (24.62%) performing the worst (Additional file 1: Table S5).

### Results of additional analyses

Sources of heterogeneity for direct comparisons were sought. Further meta-regression analysis explored each outcome regarding the degree of hypoxemia (PaO_2_/FiO_2_ at baseline), type of patient, and age (Additional file 1: Table S6). Subgroup analyses of the meta-regression results were followed to investigate the heterogeneity (Additional file 1: Table S7). Meta-regression and subgroup analyses revealed that patient type might be the main source of heterogeneity in intubation at day 28, short-term mortality, length of hospital stay, and 24-h PaO_2_/FiO_2_. And age is an influential factor in the heterogeneity of long-term mortality.

## Discussion

HFNC is a highly effective and convenient oxygen therapy, so it is vital to understand the pros and cons of different initial flow settings to avoid adverse clinical events [45]. To the best of our knowledge, there is still a lack of NMA comparing different initial flow settings for HFNC that would allow for a more precise application to clinical practice. This study investigates the efficacy and comfort of HFNC therapy with different initial flow settings in patients with AHRF. In the current NMA of the adult with AHRF, moderate quality evidence suggests that HFNC_High significantly reduced the risk of intubation and short-term mortality compared to NIV. There was no difference in comparison between the different initial flow settings of HFNC for each outcome with low or very low quality evidence. The SUCRA results showed that HFNC_High was the best intervention to reduce intubation rates and mortality. HFNC_Low had the highest probability of being the most effective in terms of comfort scores, while the HFNC_High and Mod groups had poor performance.

Oxygen therapy has always been the first-line treatment for patients with AHRF. HFNC is a novel oxygen therapy capable of delivering up to 60–70 L/min of humidified oxygen and reliably achieving and maintaining up to as high as 100% FiO_2_, which is well suited to meet the inspiratory needs of AHRF patients [46]. The flow setting of the HFNC plays a key role in its use, as the physiological effects of the HFNC are flow related. A comprehensive exploration of the various studies [36, 40, 47] and surveys [11, 48] suggests that although HFNC is widely used as oxygen therapy, the information used to guide the use of HFNC is limited and inconsistent, resulting in potential wide variation in clinical practice. Walsh and colleagues [49] designed an initial flow setting formula based on patient size, weight, and age, allowing for reasonable oxygen administration, but this is only for pediatrics. Therefore, it is essential to compare the effectiveness and comfort of different initial flow settings of HFNC for adult patients with AHRF from multiple perspectives.

What worries us the most is the invasive ventilation caused by AHRF. Acute respiratory failure progresses rapidly, often requiring mechanical ventilation in the late stages, and there is conclusive evidence of a direct relationship between invasive ventilation and the occurrence of adverse events [4, 50]. Our NMA and ranking analysis results showed that the HFNC_High group was the best strategy for reducing intubation incidence at day 28. These findings are similar to previous meta-analysis results [51, 52]. Indeed, there is proven evidence that higher flow rates (50-60L/min) significantly improve respiratory physiology in patients with AHRF. It has been identified that found that the peak tidal inspiratory flow (PTIF) required by AHRF patients can be much higher than average adults. The PTIF in patients with extremely severe hypoxemia can exceed 60 and even reach 120 L/min [45, 53]. Continuous flow delivery above PTIF produces a low level of positive pressure in the upper airway, known as the PEEP effect. Moreover, Mauri and collaborators [54] found that improvements in oxygenation, end-expiratory lung volume and lung mechanics were linearly correlated with flow rate. High initial flow settings give sufficient oxygen flow and PEEP effect to satisfy the inspiratory demand of AHRF patients, which can increase early oxygenation and decrease transpulmonary pressures, thereby preventing lung injury caused by IMV [55]. Likewise, the cumulative amplification of these physiological effects is beneficial in improving oxygenation, lowering the failure of non-invasive oxygen therapy strategies, and minimizing the danger of additional lung injury, hence preventing adverse events and complications.

Based on recent clinical practice guidelines [1, 10] and the results of several large RCTs [32–34, 40, 56], HFNC remains controversial in reducing mortality in patients with AHRF. Our NMA results showed that the HFNC_High group was significantly associated with a reduction in short-term mortality. Furthermore, HFNC_High had the highest probability of being the best treatment for short and long-term mortality as determined by SUCRA results. It is reasonable to believe that higher HFNC flow rates may be associated with positive physiological effects of improved lung protection and effective in preventing oxygen therapy failure. Consequently, the accumulated effects of reduced intubation requirements and improved oxygenation have undeniably beneficial impacts on mortality. These are perhaps the missing parts of the moderate and low flow groups. Some previous meta-analyses [52, 57] did not yield an advantage of HFNC in terms of mortality, probably due to the lack of comparison between different HFNC flow settings. The overall effect of different flow rates doped together to produce a comparison with NIV can somewhat affect the actual results.

Further meta-regression and subgroup analysis showed that the patient type was the primary source of outcomes heterogeneity. The results of the subgroup analysis found that the intubation rate and short-term mortality were significantly lower in AHRF patients without specific restrictions than with immunocompromised and COVID-19. According to the characteristics of the included studies, the cause of AHRF without specific patient type restrictions was mostly pneumonia and new-onset AHRF was more common. These patients may have the relatively less underlying disease and the effectiveness of HFNC is more readily apparent. It may explain the relatively lower occurrence of intubation and mortality associated with using HFNC. At the same time, other determinants of mortality remain highly influential. In other words, patient management, delay in intubation, ability to identify the etiology of ARF, pulmonary infection, and associated organ dysfunction are all associated with mortality [15]. Whether the initial flow strategy can overcome these strong predictors is still being determined due to the lack of adjustment for these essential confounding factors. There is a need for more sizable RCTs that take confounding factors out of the equation.

Comfort plays a key role in shaping the clinical efficacy of HFNC. As a matter of fact, for non-invasive ventilation, HFNC needs to be used for several days rather than hours. Therefore, the comfort assessment of HFNC plays a significant role in the treatment and care process. Maggiore and colleagues [47] discovered that HFNC obtained better oxygenation and enhanced comfort for the same FiO_2_ setting as NIV, which is consistent with our findings. However, with a higher flow of HFNC, comfort may suffer noticeably. The physiological study by Basile et al. [58]emphasized that HFNC with (> 60 L/min) while improving physiological outcomes, was simultaneously associated with deterioration in patient comfort. When used in clinical practice, less comfortable patients may be less tolerant of the device. Patients face conditions that can lead to unsustainable oxygen therapy or even treatment failure. Other ongoing issues are the increased noise and pressure on the esophageal wall associated with high flow rates, which can be challenging for some patients [59]. According to the NMA results, HFNC_Low had the highest probability of being the best comfort score among all the interventions. A retrospective study by Butt et al. [21] revealed that HFNC flow settings were associated with the highest mean comfort scores. Maximum comfort was observed at HFNC flow rates between 30 and 40 L/min, with a clear and gradual decrease at 50 and 60 L/min. Likewise, Roca et al. [60] observed a substantial increase in comfort in patients with AHRF receiving a somewhat lower flow of HFNC (30 [21.3–38.7] L/min). What’s more, the SUCRA results showed that the HFNC_Mod group had the best performance for ICU length of stay and demonstrated similarly to the HFNC_High group in terms of length of stay. It may imply that the moderate flow group was marginally beneficial in reducing patients’ length of stay.

Interestingly, when the flow rate is too high above the patient's PTIF, hypopharyngeal pressure rises with increasing delivered flow rate, but there is no change in FiO_2_ [20, 61]. Theologou et al. [17] reported that in patients with AHRF after cardiac surgery extubation, regardless of the initial flow of 60 L/min or 40 L/min, the incidence of treatment failure in the HFNC group was significantly lower than that in NIV group. Notably, HFNC may provide most of its physiological benefits to patients at a flow rate of 30 L/min [14]. Moreover, patients with limited potential for recruitment and a higher risk of hyperinflation experiencing higher flow rates may result in overinflation and induce lung injury [62]. These imply that patient reactions to various flows vary widely, and the optimal flow for each physiologic variable does not necessarily equate to the greatest flow (i.e., 60 L/min). Therefore, the initial flow rate setting of HFNC needs to be weighed against the patient's strategy for physiological improvement, tolerability and risk. As with intubated patients, a lower initial flow setting to minimize the risk of lung injury may be a strategy to improve the prognosis of AHRF patients treated with HFNC. As a result, the optimal initial flow setting of HFNC should begin at a moderate flow (30–40 L/min) and be modified following the patients’ actual requirements and tolerance.

Our study certainly has limitations. First of all, although the 23 included studies involved patients with AHRF, the etiology of the disease was different, which may have affected the results to some extent. Second, the definition of AHRF was different for each included study, so we could not give a uniform inclusion criterion for AHRF patients, leading to heterogeneity due to the varying degree of hypoxemia in patients. Moreover, despite our thorough search of the databases, the limited number of studies with initial flow settings at HFNC_Low and HFNC_Mod had restricted the ability to assess outcomes, especially the comfort scores. And owing to the lack of relevant RCTs for comparing HFNC initial flow settings, inconsistency testing cannot be performed. We could only perform indirect comparisons of flow rates, resulting in low or very low quality evidence. Also, the initial flow setting is not representative of ongoing flow rates, and it does indicate overall flow rates and medical staff preferences. Finally, the statistical methods and the methodological limitations of NMA must be addressed, which may lead to a different result by slight variations.

## Conclusions

After analyzing the findings presented in the 23 RCTs, we observed that high initial flow settings (50–60 L/min) performed better in reducing intubation at day 28 and short-term mortality, although comfort scores were poor. Treatment of HFNC for AHRF patients should be initiated from moderate flow rates (30–40 L/min), and individualized flow settings can make HFNC more sensible in clinical practice. Future clinical work and studies are needed to further investigate the impact of different initial flow settings of HFNC on the efficacy and comfort of patients with AHRF.

## Supplementary Information

**Additional file 1: Table S1.** PRISMA NMA checklist. **Table S2.** Search strategy. **Table S3.** Network meta-analysis: model fit details. **Table S4.** Summary of findings table and GRADE assessment of NMA. **Table S5.** Probability of each treatment to be the best. **Table S6.** Results of heterogeneity test and meta-regression for direct comparisons. **Table S7.** Subgroup analysis of the follow-up results of meta-regression. **Fig. S1.** Risk of bias summary review authors’ judgments about each risk of bias item for included RCTs. **Fig. S2.** Comparison adjusted funnel plot for the network meta-analysis. **Fig. S3.** The forest plots of pairwise meta-analysis. **Fig. S4.** Network plot of length of ICU stay, length of hospital stay, and 24-h PaO_2_/FiO_2_. **Fig. S5.** Line chart and bar chart of the surface under the cumulative ranking curve values of short-term mortality, long-term mortality, length of ICU stay, length of hospital stay, and 24-h PaO_2_/FiO_2_.

## Data Availability

Not applicable.

## Abbreviations

PRISMA: Preferred Reporting Items for Systematic Reviews and Meta-analysis
GRADE: Grades of Recommendation, Assessment, Development and Evaluation Working Group
RCT: Randomized controlled trial
OR: Odds ratio
CI: Confidence interval
CrI: Credible interval
SUCRA: Surface under the cumulative ranking curve
HFNC: High-flow nasal cannula
NIV: Non-invasive ventilation
AHRF: Acute hypoxemic respiratory failure
PaO_2_/FiO_2_ ratio: Ratio of arterial oxygen partial pressure to the fraction of inspired oxygen
PaCO_2_: Partial pressure of arterial carbon dioxide
PEEP: Positive end-expiratory pressure
NMA: Network meta-analysis
