# Supplementary material for: Comparison of the efficacy and comfort of high-flow nasal cannula with different initial flow settings in patients with acute hypoxemic respiratory failure: a systematic review and network meta-analysis
Source: J Intensive Care. 2023 May 10;11:18. doi: 10.1186/s40560-023-00667-2 (PMC10171174; doi:10.1186/s40560-023-00667-2)
Supplement: Supplementary file 1 — Additional file 1: Table S1. PRISMA NMA checklist. Table S2. Search strategy. Table S3. Network meta-analysis: model fit details. Table S4. Summary of findings table and GRADE assessment of NMA. Table S5. Probability of each treatment to be the best. Table S6. Results of heterogeneity test and meta-regression for direct comparisons. Table S7. Subgroup analysis of the follow-up results of meta-regression. Fig. S1. Risk of bias summary review authors’ judgments about each risk of bias item for included RCTs. Fig. S2. Comparison adjusted funnel plot for the network meta-analysis. Fig. S3. The forest plots of pairwise meta-analysis. Fig. S4. Network plot of length of ICU stay, length of hospital stay, and 24-h PaO2/FiO2. Fig. S5. Line chart and bar chart of the surface under the cumulative ranking curve values of short-term mortality, long-term mortality, length of ICU stay, length of hospital stay, and 24-h PaO2/FiO2. [file 40560_2023_667_MOESM1_ESM.pdf]

Comparison of the efficacy and comfort of high flow nasal cannula with different initial flow settings in adult patients with acute hypoxemic respiratory failure: A systematic review and network meta-analysis

Additional Files

Tables

Table S1 PRISMA NMA checklist

Table S2 Search strategy

Table S3 Network meta-analysis: model fit details

Table S4 Summary of findings table and GRADE assessment of NMA

Table S5 Probability of each treatment to be the best

Table S6 Results of heterogeneity test and meta-regression for direct comparisons

Table S7 Subgroup analysis of the follow-up results of meta-regression

Figures

Fig.S1: Risk of bias summary review authors' judgments about each risk of bias item for included RCTs.

Fig.S2 Comparison adjusted funnel plot for the network meta-analysis

Fig.S3 The forest plots of pairwise meta-analysis

Fig.S4 Network plot of length of ICU stay(A), length of hospital stay (B), and 24h-PaO_2/FiO_2 (C)

Fig.S5 Line chart and bar chart of the surface under the cumulative ranking curve

(SUCRA) values of short-term mortality (A), long-term mortality (B), length of ICU stay(C), length of hospital stay (D), and 24h-PaO_2/FiO_2 (E)

Additional Tables

Table S1 PRISMA NMA Checklist of Items to Include When Reporting A Systematic Review Involving a Network Meta-analysis

Section/Topic	Item #	Checklist Item	Reported on Page #
**TITLE**			
Title	1	Identify the report as a systematic review *incorporating a network meta-analysis (or related form of meta-analysis).*	1
**ABSTRACT**			
Structured summary	2	Provide a structured summary including, as applicable: **Background:** main objectives **Methods:** data sources; study eligibility criteria, participants, and interventions; study appraisal; and *synthesis methods, such as network meta-analysis.* **Results:** number of studies and participants identified; summary estimates with corresponding confidence/credible intervals; *treatment rankings may also be discussed. Authors may choose to summarize pairwise comparisons against a chosen treatment included in their analyses for brevity.* **Discussion/Conclusions:** limitations; conclusions and implications of findings. **Other:** primary source of funding; systematic review registration number with registry name.	2-3
**INTRODUCTION**			

Rationale	3	Describe the rationale for the review in the context of what is already known*, including mention of why a network meta-analysis has been conducted.*	3
Objectives	4	Provide an explicit statement of questions being addressed, with reference to participants, interventions, comparisons, outcomes, and study design (PICOS).	3-4
**METHODS**			
Protocol and registration	5	Indicate whether a review protocol exists and if and where it can be accessed (e.g., Web address); and, if available, provide registration information, including registration number.	5
Eligibility criteria	6	Specify study characteristics (e.g., PICOS, length of follow-up) and report characteristics (e.g., years considered, language, publication status) used as criteria for eligibility, giving rationale. *Clearly describe eligible treatments included in the treatment network, and note whether any have been clustered or merged into the same node (with justification).*	6-7
Information sources	7	Describe all information sources (e.g., databases with dates of coverage, contact with study authors to identify additional studies) in the search and date last searched.	5-6
Search	8	Present full electronic search strategy for at least one database, including any limits used, such that it could be repeated.	Supplementary Table S2
Study selection	9	State the process for selecting studies (i.e., screening, eligibility, included in systematic review, and,	6

		if applicable, included in the meta-analysis).	
Data collection process	10	Describe method of data extraction from reports (e.g., piloted forms, independently, in duplicate) and any processes for obtaining and confirming data from investigators.	7
Data items	11	List and define all variables for which data were sought (e.g., PICOS, funding sources) and any assumptions and simplifications made.	8
**Geometry of the network**	**S1**	Describe methods used to explore the geometry of the treatment network under study and potential biases related to it. This should include how the evidence base has been graphically summarized for presentation, and what characteristics were compiled and used to describe the evidence base to readers.	8
Risk of bias within individual studies	12	Describe methods used for assessing risk of bias of individual studies (including specification of whether this was done at the study or outcome level), and how this information is to be used in any data synthesis.	7-8
Summary measures	13	State the principal summary measures (e.g., risk ratio, difference in means). *Also describe the use of additional summary measures assessed, such as treatment rankings and surface under the cumulative ranking curve (SUCRA) values, as well as modified approaches used to present summary findings from meta-analyses.*	8-9
Planned methods of analysis	14	Describe the methods of handling data and combining results of studies for each network meta-analysis. This should include, but	9

		not be limited to:<br>• *Handling of multi-arm trials;*<br>• *Selection of variance structure;*<br>• *Selection of prior distributions in Bayesian analyses; and*<br>• *Assessment of model fit.*	
**Assessment of Inconsistency**	**S2**	Describe the statistical methods used to evaluate the agreement of direct and indirect evidence in the treatment network(s) studied. Describe efforts taken to address its presence when found.	9
Risk of bias across studies	15	Specify any assessment of risk of bias that may affect the cumulative evidence (e.g., publication bias, selective reporting within studies).	8
Additional analyses	16	Describe methods of additional analyses if done, indicating which were pre-specified. This may include, but not be limited to, the following:<br>• Sensitivity or subgroup analyses;<br>• Meta-regression analyses;<br>• *Alternative formulations of the treatment network; and*<br>• *Use of alternative prior distributions for Bayesian analyses (if applicable).*	10
**RESULTS†**			
Study selection	17	Give numbers of studies screened, assessed for eligibility, and included in the review, with reasons for exclusions at each stage, ideally with a flow diagram.	10

**Presentation of network structure**	**S3**	Provide a network graph of the included studies to enable visualization of the geometry of the treatment network.	13
**Summary of network geometry**	**S4**	Provide a brief overview of characteristics of the treatment network. This may include commentary on the abundance of trials and randomized patients for the different interventions and pairwise comparisons in the network, gaps of evidence in the treatment network, and potential biases reflected by the network structure.	13
Study characteristics	18	For each study, present characteristics for which data were extracted (e.g., study size, PICOS, follow-up period) and provide the citations.	11
Risk of bias within studies	19	Present data on risk of bias of each study and, if available, any outcome level assessment.	12
Results of individual studies	20	For all outcomes considered (benefits or harms), present, for each study: 1) simple summary data for each intervention group, and 2) effect estimates and confidence intervals. *Modified approaches may be needed to deal with information from larger networks.*	12-13
Synthesis of results	21	Present results of each meta-analysis done, including confidence/credible intervals. *In larger networks, authors may focus on comparisons versus a particular comparator (e.g. placebo or standard care), with full findings presented in an appendix. League tables and forest plots may be considered to summarize pairwise comparisons.* If additional summary measures were explored (such as treatment rankings), these should	13-16

		also be presented.	
**Exploration for inconsistency**	**S5**	Describe results from investigations of inconsistency. This may include such information as measures of model fit to compare consistency and inconsistency models, *P* values from statistical tests, or summary of inconsistency estimates from different parts of the treatment network.	12
Risk of bias across studies	22	Present results of any assessment of risk of bias across studies for the evidence base being studied.	12
Results of additional analyses	23	Give results of additional analyses, if done (e.g., sensitivity or subgroup analyses, meta-regression analyses, *alternative network geometries studied, alternative choice of prior distributions for Bayesian analyses,* and so forth).	16
**DISCUSSION**			
Summary of evidence	24	Summarize the main findings, including the strength of evidence for each main outcome; consider their relevance to key groups (e.g., healthcare providers, users, and policy-makers).	17
Limitations	25	Discuss limitations at study and outcome level (e.g., risk of bias), and at review level (e.g., incomplete retrieval of identified research, reporting bias). *Comment on the validity of the assumptions, such as transitivity and consistency. Comment on any concerns regarding network geometry (e.g., avoidance of certain comparisons).*	22-23
Conclusions	26	Provide a general interpretation of the results in the context of other evidence, and implications for future research.	23

**FUNDING**			
Funding	27	Describe sources of funding for the systematic review and other support (e.g., supply of data); role of funders for the systematic review. This should also include information regarding whether funding has been received from manufacturers of treatments in the network and/or whether some of the authors are content experts with professional conflicts of interest that could affect use of treatments in the network.	24

PICOS = population, intervention, comparators, outcomes, study design.
* Text in italics indicateS wording specific to reporting of network meta-analyses that has been added to guidance from the PRISMA statement.
† Authors may wish to plan for use of appendices to present all relevant information in full detail for items in this section.

Table S2 Search strategy

1. *PubMed (Performed on October 10th, 2022)*

Number	Searched for
#1	"Respiratory Insufficiency"[mh] OR "Hypoxia"[mh] OR hypoxaemic acute respiratory failure*[tiab] OR acute hypoxaemic respiratory failure*[tiab] OR acute hypoxaemic respiratory failure*[tiab] OR acute hypoxemic respiratory failure*[tiab] OR hypoxemic acute respiratory failure*[tiab] OR respiratory failure*[tiab] OR failure respiratory*[tiab] OR hypoxemic respiratory failure*[tiab] OR acute respiratory distress*[tiab] adult respiratory distress syndrome*[tiab] OR respiratory depression*[tiab] OR ventilatory depression OR AHRF[tiab] OR ARDS[tiab]
#2	high flow nasal cannula*[tiab] OR HFNC[tiab] OR HHFNC[tiab] OR high flow nasal cannula therapy*[tiab] OR nasal high flow*[tiab] OR high flow nasal cannula*[tiab] OR high flow nasal oxygen*[tiab] OR HFNO[tiab] OR HFO[tiab] OR high velocity nasal insufflation*[tiab] OR high flow oxygen*[tiab] OR NHF[tiab]
#3	("Randomized Controlled Trials as Topic"[mh] OR "Randomized Controlled Trial"[pt] OR "Controlled Clinical Trial"[pt] OR Randomized[tiab] OR Randomly[tiab] OR Trial[tiab] OR Groups[tiab]) NOT ("Animals"[mh] NOT "Humans"[mh])
#4	#1 and #2 and #3

2. *Embase (Performed on October 10th, 2022)*

Number	Searched for
#1	'respiratory failure'/exp
#2	'hypoxia'/exp
#3	#1 OR #2
#4	'acute hypoxemic respiratory failure':ti,ab,kw OR 'acute hypoxaemic respiratory failure':ti,ab,kw OR 'failure, respiratory':ti,ab,kw OR 'hypoxemic respiratory failure':ti,ab,kw OR 'adult respiratory distress syndrome':ti,ab,kw OR 'respiration depression':ti,ab,kw OR 'ventilatory depression':ti,ab,kw OR AHRF:ti,ab,kw OR ARDS:ti,ab,kw
#5	#3 OR #4
#6	'high flow nasal cannula therapy'/exp
#7	'high flow nasal cannula':ti,ab,kw OR HFNC:ti,ab,kw OR 'nasal high flow':ti,ab,kw OR 'high flow nasal oxygen':ti,ab,kw OR hfno:ti,ab,kw OR 'high-velocity nasal insufflation':ti,ab,kw OR 'high flow oxygen':ti,ab,kw OR NHF:ti,ab,kw OR 'humidified high flow nasal cannula therapy':ti,ab,kw OR HHFNC:ti,ab,kw OR HFO:ti,ab,kw OR HFOT:ti,ab,kw
#8	#6 OR #7
#9	'randomized controlled trial'/exp
#10	'controlled clinical trial (topic)'/exp
#11	randomly:ti,ab,kw OR randomized:ti,ab,kw OR groups:ti,ab,kw OR trial:ti,ab,kw
#12	#9 OR #10 OR #11
#13	#5 AND #8 AND #12

3. Web of Science (Performed on October 10th, 2022)

Number	Searched for
#1	TS=(Respiratory Insufficiency* OR hypoxia* OR Hypoxaemic Acute Respiratory Failure* OR Acute Hypoxaemic Respiratory Failure* OR Acute Hypoxemic Respiratory Failure* OR Hypoxemic Acute Respiratory Failure* OR Respiratory Failure* OR Failure, Respiratory OR Hypoxemic Respiratory Failure OR Acute Respiratory Distress* OR Respiratory Depression OR Ventilatory Depression OR AHRF OR ARDS)
#2	TS= (High Flow Nasal Cannula* OR HFNC OR HHFNC OR High flow nasal cannula therapy* OR Nasal high flow* OR High-Flow Nasal Cannula* OR High Flow Nasal Oxygen* OR HFNO OR HFO OR HFOT OR High-Velocity Nasal Insufflation* OR High flow oxygen* OR NHF)
#3	TS=((Randomized Controlled Trials* OR Controlled Clinical Trials* OR randomized OR randomly OR groups) NOT Animals NOT Humans))
#4	#1 AND #2 AND #3

4. Cochrane Library (CENTRAL) (Performed on October 10th, 2022)

Number	Searched for
#1	MeSH descriptor: [Respiratory Insufficiency] explode all trees
#2	MeSH descriptor: [Respiratory Distress Syndrome] explode all trees
#3	(Respiratory Failure*):ti,ab,kw OR (Acute Hypoxemic Respiratory Failure*):ti,ab,kw OR (Hypoxemic Acute Respiratory Failure*):ti,ab,kw
#4	(Respiratory Depression*):ti,ab,kw OR (Ventilatory Depression*):ti,ab,kw OR (AHRF):ti,ab,kw OR (ARDS):ti,ab,kw
#5	#1 OR #2 OR #3 OR #4
#6	(High Flow Nasal Cannula*):ti,ab,kw OR (High flow nasal cannula therapy*):ti,ab,kw OR (HFNC):ti,ab,kw OR (Nasal high flow*):ti,ab,kw OR (NHF):ti,ab,kw
#7	(High Flow Nasal Oxygen*):ti,ab,kw OR (HFNO):ti,ab,kw OR (HHFNC):ti,ab,kw OR (HFO):ti,ab,kw OR (HFOT):ti,ab,kw
#8	(High-Velocity Nasal Insufflation*):ti,ab,kw OR (High flow oxygen):ti,ab,kw
#9	#6 OR #7 OR #8
#10	MeSH descriptor: [Randomized Controlled Trials as Topic] explode all trees
#11	(controlled clinical trials*):ti,ab,kw OR (randomized):ti,ab,kw OR (randomly):ti,ab,kw OR (groups):ti,ab,kw NOT (animal*):ti,ab,kw
#12	#10 OR #11
#13	#5 AND #9 AND #12

5. China National Knowledge Infrastructure database (CNKI) (Performed on October 10th, 2022)

Number	Searched for
#1	SU%=‘呼吸衰竭’ OR SU%=‘呼吸窘迫’
#2	TKA='急性缺氧性呼吸衰竭' OR TKA='缺氧性呼吸衰竭' OR TKA= '急性呼吸衰竭' OR TKA= '成人呼吸窘迫综合征' OR TKA = '呼吸抑制' OR TKA = 'AHRF' OR TKA = 'ARDS'
#3	#1 OR #2
#4	TKA = '高流量鼻导管通气' OR TKA = '高流量鼻导管吸氧' OR TKA = '高流量鼻导管氧疗' OR TKA = '经鼻高流量氧疗' OR TKA = '经鼻高流量湿化氧疗' OR TKA = '加温湿化高流量鼻导管' OR TKA = '湿化高流量鼻导管通气' OR TKA = '鼻导管湿化氧疗 ' OR TKA = ' HFNC' OR TKA = 'HFNO' OR TKA ='NHF'
#5	SU%='随机对照试验' OR （TKA = '对照试验研究' OR TKA = '随机对照研究' OR TKA = '随机对照' OR TKA = '随机分组' OR TKA = '随机选择'） NOT（TKA = '动物实验' OR TKA = '动物模型'）
#6	#3 AND #4 AND #5

Table S3 Network meta-analysis: model fit details

	Fixed-effects model (consistency)	Random-effects model (consistency)	Inconsistency model
**Intubation at day 28**			
Dbar	54.55	34.18	32.73
pD	18.98	27.72	32.72
DIC	73.52	**61.91***	65.45
I^2	43%	9%	5%
**Short-term mortality**			
Dbar	33.60	27.35	26.49
pD	16.06	21.25	26.48
DIC	49.66	**48.59***	52.97
I^2	26%	9%	6%
**Long-term mortality**			
Dbar	16.59	12.27	12.26
pD	9.11	11.28	12.26
DIC	25.71	**23.55***	24.51
I^2	34%	12%	10%
**Comfort scores**			
Dbar	12.74	10.80	10.03
pD	8.03	9.44	10.02
DIC	20.77	**19.54***	20.05
I^2	29%	17%	10%
**Length of ICU stay**			
Dbar	35.97	26.69	24.06
pD	14.97	20.11	24.05
DIC	50.95	**46.80***	48.12
I^2	36%	14%	4%
**Length of hospital stay**			
Dbar	51.94	24.31	24.05
pD	14.98	21.30	24.05
DIC	66.92	**45.61***	48.10
I^2	56%	5%	4%
**24h-PaO_2/FiO_2**			
Dbar	1015.96	16.14	16.03
pD	43.67	15.90	16.03
DIC	1059.63	**32.02***	32.06
I^2	99%	7%	6%

Dbar: posterior mean of deviance; DIC: deviance information criteria; pD: effective number of parameters or leverage.

* In general, the smaller the DIC, the better the model fit is indicated. Therefore, random effects models were chosen in our NMA.

Table S4A Summary of findings table and GRADE assessment of NMA for the intubation at day 28

**Outcome: Intubation at day 28**<br>**Intervention: Various interventions**<br>**Follow-up period: 0 to 28 days**							
Total studies: 16 RCTs<br>Total participants: 3,976	Relative effect<br>(95% CrI)	Anticipated absolute effect (95% CrI)			Certainty of evidence	Rank based on SUCRA	Reason(s) for downgrading
		Without intervention	With intervention	Difference			
HFNC_High vs NIV<br>(11 RCTs, 3510 participants)	OR 0.72<br>(0.56 to 0.93)<br>Network estimate	418per 1000*	301 per 1000	117 fewer<br>(184 fewer to 29 fewer)	⊕⊕⊕◯<br>Moderate	1<br>(73.04%)	Heterogeneity[1]
HFNC_Mod vs NIV<br>(3 RCTs, 320 participants)	OR 0.69<br>(0.40 to 1.17)<br>Network estimate	418 per 1000*	288 per 1000	130 fewer<br>(251 fewer to 71 more)	⊕⊕⊕◯<br>Moderate	2<br>(57.52%)	Imprecision[2]
HFNC_Low vs NIV<br>(2 RCTs, 146 participants)	OR 0.78<br>(0.12 to 5.11)<br>Network estimate	418 per 1000*	326 per 1000	92 fewer<br>(367 fewer to 1717 more)	⊕⊕◯◯<br>Low	3<br>(44.53%)	Imprecision[2]<br>Reporting bias[3]
NIV	Reference comparator	NE	NE	NE	Reference comparator	4<br>(22.90%)	Reference comparator
HFNC_High vs HFNC_Mod<br>(No direct comparison)	OR 0.99<br>(0.44 to 2.23)<br>Network estimate	418 per 1000*	413 per 1000	5 fewer<br>(234 fewer to 514 more)	⊕◯◯◯<br>Very Low	NE	Imprecision[2]<br>Inconsistency[4]<br>Indirectness[5]
HFNC_High vs HFNC_Low<br>(No direct comparison)	OR 0.78<br>(0.31 to 1.97)<br>Network estimate	418 per 1000*	326 per 1000	92 fewer<br>(288 fewer to 405 more)	⊕◯◯◯<br>Very Low	NE	Imprecision[2]<br>Inconsistency[4]<br>Indirectness[5]

HFNC_Low vs HFNC_Mod (No direct comparison)	OR 1.27 (0.39 to 4.04) Network estimate	418 per 1000*	530 per 1000	112 more (254 fewer to 1270 more)	⊕◯◯◯ Very Low	NE	Imprecision[2] Inconsistency[4] Indirectness[5]

HFNC_High: High flow nasal cannula treatment with initial flow above 50L/min; HFNC_Mod: High flow nasal cannula treatment with initial flow between 35-50L/min; HFNC_Low: High flow nasal cannula treatment with initial flow below 35L/min; NIV: Noninvasive ventilation; OR: Odds rat io; CrI: Credible interval; SUCRA: Surface under the cumulative ranking; NE: Not estimate.

GRADE Working Group grades of evidence
High quality: Further research is very unlikely to change our confidence in the estimate of effect
Moderate quality: Further research is likely to have an important impact on our confidence in the estimate of effect and may change the estimate
Low quality: Further research is very likely to have an important impact on our confidence in the estimate of effect and is likely to change the estimate
Very low quality: We are very uncertain about the estimate
The corresponding risk (and its 95% credible interval) is based on the assumed risk in the comparison group and the relative effect of the intervention (and its 95% CrI)
The estimated value of between-study variance for the network meta-analysis is 0.123.
* Based on assumed control risk of intubation at day 28 of 41.8% (corresponding to a pooled 41.8% rate of intubation occurrence within 28 days receiving NIV treatment in included studies).

[1] Downgraded for serious heterogeneity, with prediction interval extends into clinically important effects in both directions.

[2] Downgraded for serious imprecision, because confidence interval extends into clinically important effects in both directions.

[3] Downgraded for serious reporting bias.

[4] Downgraded all indirect evidence for serious inconsistency. Since the comparisons between different initial flow settings of HFNC are all indirect comparisons, the network meta-analysis does not have a closed loop and cannot perform the inconsistency model test.

[5] Downgraded for indirectness caused by intransitivity.

Table S4B Summary of findings table and GRADE assessment of NMA for the short-term mortality

**Outcome: Short-term mortality**<br>**Intervention: Various interventions**<br>**Follow-up period: 0 to 30 days**							
Total studies: 14 RCTs<br>Total participants: 3,905	Relative effect (95% CrI)	Anticipated absolute effect (95% CrI)			Certainty of evidence	Rank based on SUCRA	Reason(s) for downgrading
		Without intervention	With intervention	Difference			
HFNC_High vs NIV (11 RCTs, 3545 participants)	OR 0.81 (0.69 to 0.96) Network estimate	241 per 1000	195 per 1000	46 fewer (75 fewer to 10 fewer)	⊕⊕⊕◯ Moderate	1 (82.74%)	Heterogeneity[1]
HFNC_Mod vs NIV (2 RCTs, 260 participants)	OR 0.85 (0.44 to 1.63) Network estimate	241 per 1000	204 per 1000	37 fewer (135 fewer to 152 more)	⊕⊕⊕◯ Moderate	2 (60.22%)	Imprecision[2]
HFNC_Low vs NIV (1 RCT, 100 participants)	OR 1.41 (0.64 to 3.15) Network estimate	241 per 1000	339 per 1000	98 more (87 fewer to 518 more)	⊕◯◯◯ Very Low	4 (19.54%)	Imprecision[2]<br>Risk of bias[3]
NIV	Reference comparator	NE	NE	NE	Reference comparator	3 (37.49%)	Reference comparator
HFNC_High vs HFNC_Mod (No direct comparison)	OR 0.86 (0.38 to 2.02) Network estimate	241 per 1000	207 per 1000	34 fewer (173 fewer to 246 more)	⊕◯◯◯ Very Low	NE	Imprecision[2]<br>Inconsistency[4]<br>Indirectness[5]
HFNC_High vs HFNC_Low (No direct comparison)	OR 0.52 (0.19 to 1.37) Network estimate	241 per 1000	125 per 1000	116 fewer (195 fewer to 89 more)	⊕◯◯◯ Very Low	NE	Imprecision[2]<br>Inconsistency[4]<br>Indirectness[5]

HFNC_Low vs HFNC_Mod (No direct comparison)	OR 1.69 (0.49 to 5.83) Network estimate	241 per 1000	407 per 1000	166 more (122 fewer to 1164 more)	⊕○○○ Very Low	NE	Imprecision[2] Inconsistency[4] Indirectness[5]

HFNC_High: High flow nasal cannula treatment with initial flow above 50L/min; HFNC_Mod: High flow nasal cannula treatment with initial flow between 35-50L/min; HFNC_Low: High flow nasal cannula treatment with initial flow below 35L/min; NIV: Noninvasive ventilation; OR: Odds ratio; CrI: Credible interval; SUCRA: Surface under the cumulative ranking; NE: Not estimate.

GRADE Working Group grades of evidence:
High quality: Further research is very unlikely to change our confidence in the estimate of effect
Moderate quality: Further research is likely to have an important impact on our confidence in the estimate of effect and may change the estimate
Low quality: Further research is very likely to have an important impact on our confidence in the estimate of effect and is likely to change the estimate
Very low quality: We are very uncertain about the estimate
The corresponding risk (and its 95% credible interval) is based on the assumed risk in the comparison group and the relative effect of the intervention (and its 95% CrI)
The estimated value of between-study variance for the network meta-analysis is 0.064.
* Based on assumed control risk of short-term mortality of 24.1% (corresponding to a pooled 24.1% rate of mortality within 30 days receiving NIV treatment in included studies).

[1] Downgraded for serious heterogeneity, with prediction interval extends into clinically important effects in both directions.
[2] Downgraded for serious imprecision, because confidence interval extends into clinically important effects in both directions.
[3] Downgraded for serious risk of bias, because the included RCT was not blinded to patients and caregivers and had risk of reporting bias.
[4] Downgraded all indirect evidence for serious inconsistency. Since the comparisons between different initial flow settings of HFNC are all indirect comparisons, the network meta-analysis does not have a closed loop and cannot perform the inconsistency model test.
[5] Downgraded for indirectness caused by intransitivity.

Table S4C Summary of findings table and GRADE assessment of NMA for the long-term mortality

Outcome: Long-term mortality
Intervention: Various interventions
Follow-up period: 0 to 90 days

Total studies: 6 RCTs<br>Total participants: 1,485	Relative effect<br>(95% CrI)	Anticipated absolute effect (95% CrI)			Certainty of evidence	Rank based on SUCRA	Reason(s) for downgrading
		Without intervention	With intervention	Difference			
HFNC_High vs NIV<br>(3 RCTs, 1004 participants)	OR 0.59<br>(0.31 to 1.11)<br>Network estimate	253 per 1000	149 per 1000	104 fewer<br>(175 fewer to 28 more)	⊕⊕◯◯<br>Low	1<br>(67.08%)	Imprecision[1]
HFNC_Mod vs NIV<br>(2 RCTs, 383 participants)	OR 0.66<br>(0.16 to 2.77)<br>Network estimate	253 per 1000	167 per 1000	86 fewer<br>(213 fewer to 447 more)	⊕⊕◯◯<br>Low	2<br>(58.16%)	Imprecision[1]<br>Risk of bias[2]
HFNC_Low vs NIV<br>(1 RCT, 98 participants)	OR 0.63<br>(0.10 to 3.92)<br>Network estimate	253 per 1000	159 per 1000	94 fewer<br>(227 fewer to 738 more)	⊕◯◯◯<br>Low	3<br>(51.66%)	Imprecision[1]<br>Indirectness[3]
NIV	Reference comparator	NE	NE	NE	Reference comparator	4<br>(23.11%)	Reference comparator
HFNC_High vs HFNC_Mod<br>(No direct comparison)	OR 0.81<br>(0.22 to 2.95)<br>Network estimate	253 per 1000	205 per 1000	48 fewer<br>(197 fewer to 493 more)	⊕◯◯◯<br>Very Low	NE	Imprecision[1]<br>Indirectness[3]<br>Inconsistency[4]
HFNC_High vs HFNC_Low<br>(No direct comparison)	OR 0.92<br>(0.09 to 9.36)<br>Network estimate	253 per 1000	232 per 1000	21 fewer<br>(230 fewer to 2115 more)	⊕◯◯◯<br>Very Low	NE	Imprecision[1]<br>Indirectness[3]<br>Inconsistency[4]

HFNC_Low vs HFNC_Mod (No direct comparison)	OR 0.88 (0.08 to 9.68) Network estimate	253 per 1000	223 per 1000	30 fewer (233 fewer to 2196 more)	⊕○○○ Very Low	NE	Imprecision[1] Indirectness[3] Inconsistency[4]

HFNC_High: High flow nasal cannula treatment with initial flow above 50L/min; HFNC_Mod: High flow nasal cannula treatment with initial flow between 35-50L/min; HFNC_Low: High flow nasal cannula treatment with initial flow below 35L/min; NIV: Noninvasive ventilation; OR: Odds ratio; CrI: Credible interval; SUCRA: Surface under the cumulative ranking; NE: Not estimate.

GRADE Working Group grades of evidence:
High quality: Further research is very unlikely to change our confidence in the estimate of effect
Moderate quality: Further research is likely to have an important impact on our confidence in the estimate of effect and may change the estimate
Low quality: Further research is very likely to have an important impact on our confidence in the estimate of effect and is likely to change the estimate
Very low quality: We are very uncertain about the estimate
The corresponding risk (and its 95% credible interval) is based on the assumed risk in the comparison group and the relative effect of the intervention (and its 95% CrI)
The estimated value of between-study variance for the network meta-analysis is 0.354.
* Based on assumed control risk of long-term mortality of 25.3% (corresponding to a pooled 25.3% rate of mortality within 90 days receiving NIV treatment in included studies).

[1] Downgraded for serious imprecision, because confidence interval extends into clinically important effects in both directions.
[2] Downgraded for serious risk of bias, because the included RCT was not blinded to patients and caregivers and had risk of reporting bias.
[3] Downgraded for indirectness caused by intransitivity.
[4] Downgraded all indirect evidence for serious inconsistency. Since the comparisons between different initial flow settings of HFNC are all indirect comparisons, the network meta-analysis does not have a closed loop and cannot perform the inconsistency model test.

Table S5 Probability of each treatment to be the best*

Intervention / Outcome	HFNC_High	HFNC_Mod	HFNC_Low	NIV
Intubation at day 28	**73.04%**	57.52%	44.53%	22.90%
Short-term mortality	**82.74%**	60.22%	19.54%	37.49%
Long-term mortlity	**67.08%**	58.16%	51.66%	23.11%
Comfort scores	32.07%	21.62%	**88.37%**	57.94%
Length of ICU stay	40.59%	**78.29%**	53.62%	27.50%
Length of hospital stay	**60.28%**	55.21%	59.90%	24.62%
24h-PaO_2/FiO_2	38.91%	**70.72%**	40.93%	49.44%

* Values highlighted in **bold** indicate the treatment with the highest probability of being the best for the outcome.

Table S6 Results of heterogeneity test and meta-regression for direct comparisons

Outcomes/Covariate	No. of studies	Test of heterogeneity		P value of Egger's test	Meta-regression	
		I-square	Tau-square[†]		Median coefficient[*] (95% CI)	P value
Intubation at day 28	16	58.8%	0.17	0.292		
PaO_2/FiO_2 at baseline		0	0.19		0.002(-0.005 to 0.011)	0.557
Type of patients		57.91%	0.07		**-0.670(-1.171 to -0.169)**	**0.031**
Age		0	0.20		0.003(-0.062 to 0.071)	0.932
Short-term mortality	14	34.8%	0.04	0.016		
PaO_2/FiO_2 at baseline		0	0.06		0.002(-0.005 to 0.009)	0.544
Type of patients		100%	0		**-0.525(-1.008 to -0.042)**	**0.009**
Age		0	0.08		0.002(-0.061 to 0.064)	0.956
Long-term mortality	6	55.6%	0.22	0.257		
PaO_2/FiO_2 at baseline		0	0.23		-0.006(-0.0321 to 0.020)	0.588
Type of patients		0	0.35		-0.470(-2.643 to 1.701)	0.818
Age		65.28%	0.07		**0.064(0.0030 to 0.122)**	**0.031**
Comfort scores	5	81.7%	1.24	0.592		
PaO_2/FiO_2 at baseline		0	1.53		0.005(-0.023 to 0.033)	0.721
Type of patients		0	1.86		-1.630(-4.997 to 1.731)	0.588
Age		0	1.67		0.034(-0.223 to 0.290)	0.795
Length of ICU stay	12	57.6%	0.28	0.969		
PaO_2/FiO_2 at baseline		0	0.34		0.001(-0.014 to 0.015)	0.952
Type of patients		0	0.71		-1.600(-4.234 to 1.034)	0.688
Age		0	0.52		-0.028(-0.129 to 0.074)	0.595
Length of hospital stay	12	71.1%	2.86	0.949		
PaO_2/FiO_2 at baseline		0	3.37		-0.008(-0.048 to 0.032)	0.692
Type of patients		100%	0		**-4.429(-6.177 to -2.682)**	**<0.0001**
Age		0	3.29		-0.067(-0.317 to 0.184)	0.601
24h-PaO_2/FiO_2	8	99.3%	1190.67	0.024		
PaO_2/FiO_2 at baseline		32.08%	808.67		**0.499(0.019 to 0.976)**	**0.041**
Type of patients		69.33%	365.15		**-53.915 (-103.723 to -4.107)**	**0.002**
Age		0	1235.51		2.846(-3.279 to 8.971)	0.363

[*] If the 95% CI does not cross 0 and the P-value is less than 0.05, this means that the covariates exhibit significant coefficients in the interaction model (values highlighted in **bold**).

[†] The tau-square calculated from the meta-regression represents the estimate of residual heterogeneity.

Table S7 Subgroup analysis of the follow-up results of meta-regression

Outcome/Subgroup	No. of studies	Results	P value	I-square	Tau-square	Difference between subgroups
**Intubation at day 28**	16	OR 0.72 (95%CI 0.55 to 0.95)	0.02	58.8%	0.17	
Type of patients						0.002
COVID-19 patients	4	OR 0.92 (95%CI 0.53 to 1.61)	0.62	75%	0.06	
Immunocompromised patients	5	OR 0.96 (95%CI 0.76 to 1.21)	0.82	26%	0.73	
No specific restrictions	7	OR 0.47 (95%CI 0.35 to 0.63)	<0.0001	0	0.26	
**Short-term mortality**	14	OR 0.83 (95%CI 0.71 to 0.97)	0.02	34.8%	0.04	
Type of patients						0.006
COVID-19 patients	5	OR 0.83 (95%CI 0.64 to 1.06)	0.12	13.7%	<0.001	
Immunocompromised patients	4	OR 1.02 (95%CI 0.81 to 1.30)	0.86	0	0	
No specific restrictions	5	OR 0.46 (95%CI 0.28 to 0.77)	<0.0001	19.4%	0.11	
**Long-term mortality**	6	OR 0.64 (95%CI 0.38 to 1.07)	0.09	55.6%	0.22	
Age						0.200
Above 60 years old	4	OR 0.75 (95%CI 0.41 to 1.38)	0.34	61.1%	0.23	
Below 60 years old	2	OR 0.39 (95%CI 0.18 to 0.86)	0.02	0	0	
**Length of hospital stay**	12	MD -1.10 (95%CI -2.37 to 0.18)	0.09	71.1%	2.85	
Type of patients						<0.0001
COVID-19 patients	4	MD 0.41 (95%CI -0.99 to 1.83)	0.60	31.3%	0.57	
Immunocompromised patients	4	MD -0.89 (95%CI -2.17 to 0.39)	0.18	0	0	
Cardiothoracic surgery patients	1	MD 0.40 (95%CI -0.82 to 1.62)	0.52	NE	NE	
No specific restrictions	3	MD -4.03 (95%CI -5.28 to -2.78)	<0.0001	0	0	
**24-PaO_2/FiO_2**	8	MD 7.76 (95%CI -16.94 to 32.45)	0.53	99.3%	1190.67	
PaO_2/FiO_2 at baseline						0.155
Above 150	2	MD 37.87	0.14	99.6%	1305.59	

		(95%CI -12.30 to 88.05)				
Below 150	6	MD -2.86 (95%CI -28.09 to 22.38)	<0.0001	94.4%	890.34	
Type of patients						<0.0001
COVID-19 patients	2	MD -41.61 (95%CI -74.98 to -8.25)	0.01	68.5%	400.67	
Immunocompromised patients	1	MD 26.90 (95%CI 26.64 to 27.16)	<0.0001	NE	NE	
Cardiothoracic surgery patients	2	MD 45.08 (95%CI 5.66 to 84.50)	0.03	90.5%	738.75	
AIS	1	MD 12.20 (95%CI 6.39 to 18.01)	<0.0001	NE	NE	
No specific restrictions	2	MD 6.52 (95%CI -1.86 to 14.89)	0.13	0	0	

OR: Odds ratio; CI: Confidence interval; MD: Mean difference; NE: Not estimated; COVID-19: the novel coronavirus disease 2019; AIS: Acute ischemic stroke.

Additional Figures

Fig.S1 Risk of bias summary review authors' judgments about each risk of bias item for included RCTs.

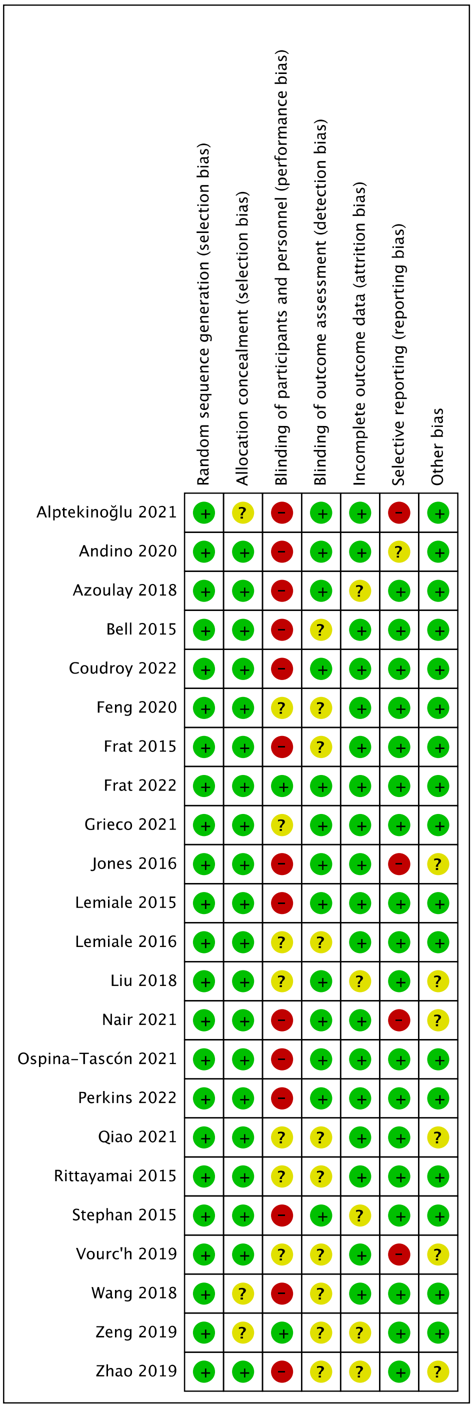

Fig.S2 Comparison adjusted funnel plot for the network meta-analysis

A. Intubation at day 28

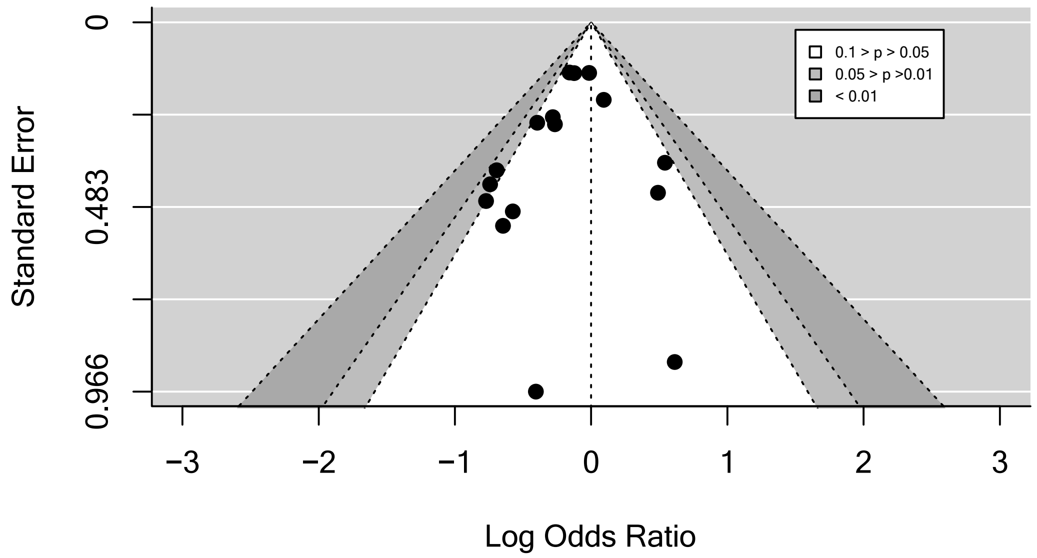

B. Short-term mortality

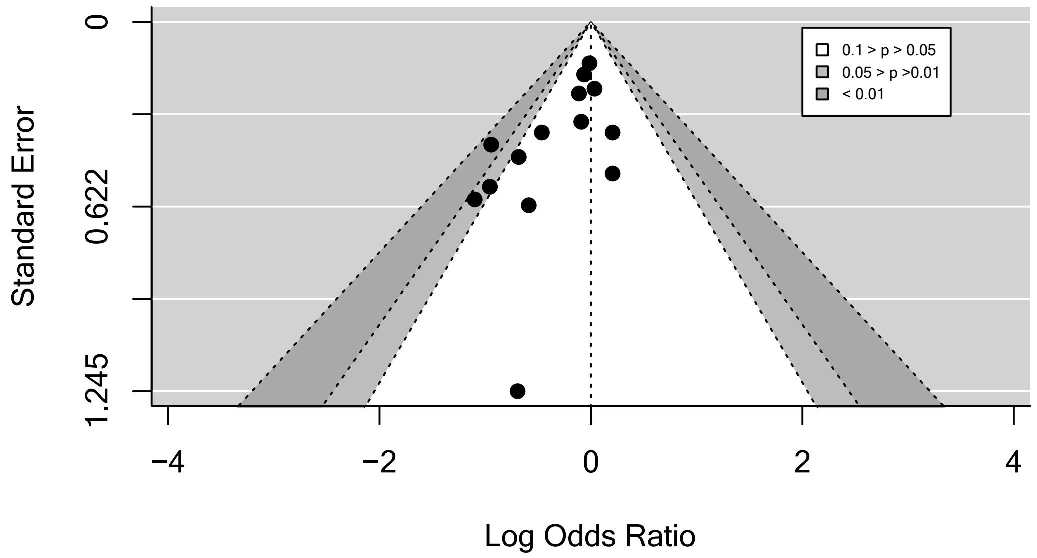

C. Length of ICU stay

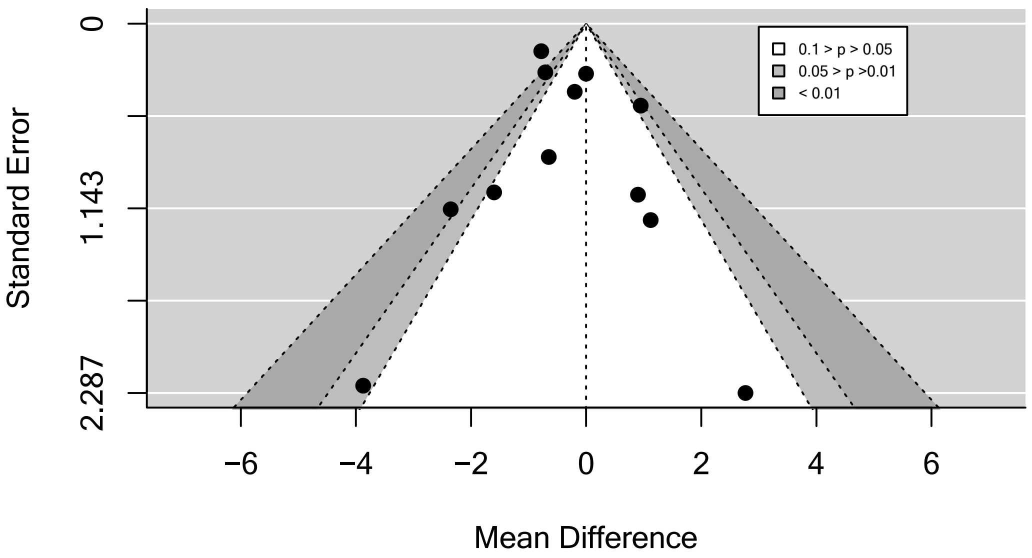

D. Length of hospital stay

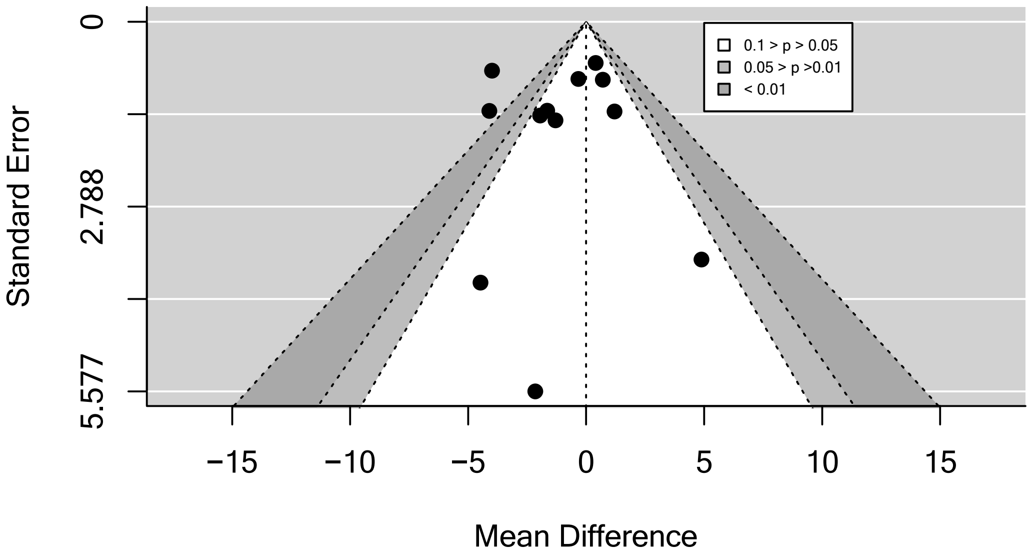

Fig.S3 The forest plots of pairwise meta-analysis

A. Intubation at day 28

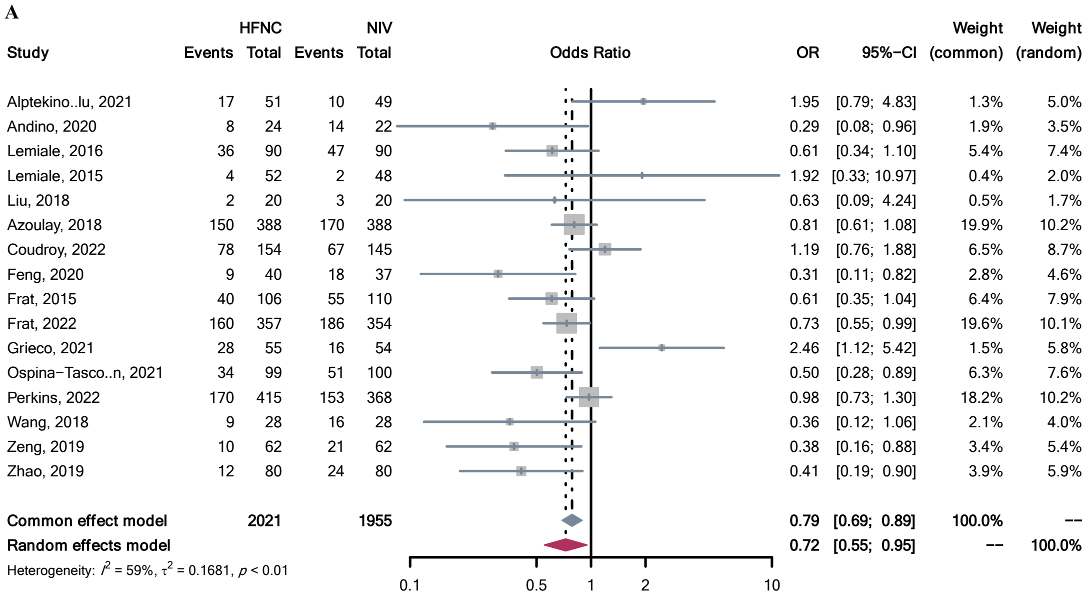

B. Short-term mortality

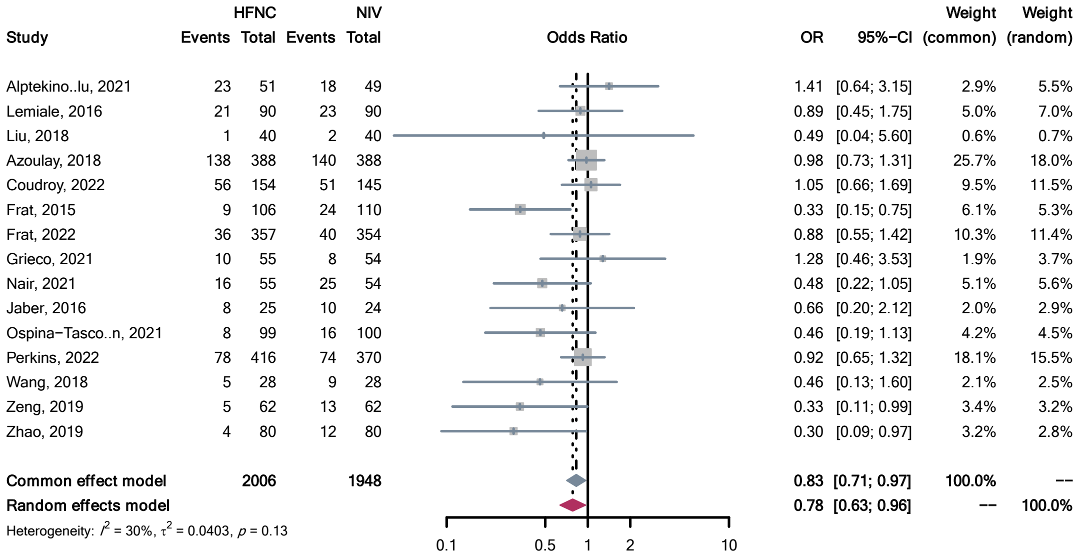

C. Long-term mortality

Study	HFNC Events	HFNC Total	NIV Events	NIV Total	OR	95%−CI	Weight (common)	Weight (random)
Qiao, 2021	2	50	3	48	0.62	[0.10; 3.92]	2.5%	6.3%
Jones, 2016	35	165	24	138	1.28	[0.72; 2.28]	17.6%	22.6%
Liu, 2018	6	40	15	40	0.29	[0.10; 0.86]	10.9%	13.2%
Feng, 2020	6	40	9	37	0.55	[0.17; 1.73]	6.8%	12.3%
Frat, 2015	13	106	31	110	0.36	[0.17; 0.73]	22.8%	19.6%
Frat, 2022	48	357	53	354	0.88	[0.58; 1.34]	39.4%	26.0%
**Common effect model**		**758**		**727**	**0.74**	**[0.56; 0.97]**	**100.0%**	**--**
**Random effects model**					**0.64**	**[0.38; 1.07]**	**--**	**100.0%**

Heterogeneity: $I^2 = 56\%$, $\tau^2 = 0.2180$, $p = 0.05$

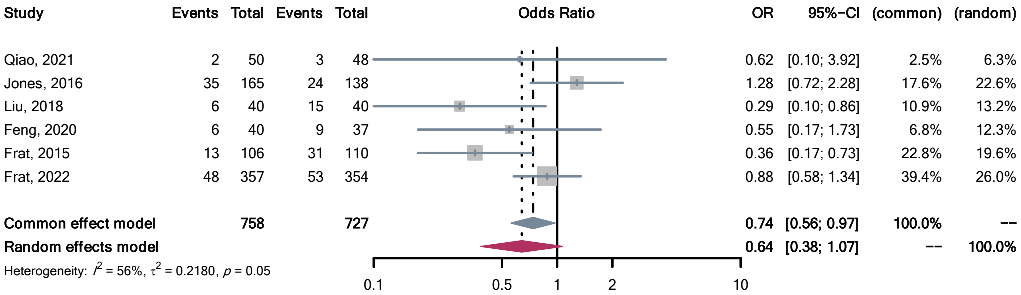

D. Comfort scores

Study	HFNC Total	HFNC Mean	HFNC SD	NIV Total	NIV Mean	NIV SD	MD	95%−CI	Weight (common)	Weight (random)
Alptekino..lu, 2021	51	5.60	5.30	49	4.90	6.90	0.70	[−1.72; 3.12]	1.1%	11.7%
Bell, 2015	48	3.60	0.80	51	3.00	1.50	0.60	[0.13; 1.07]	28.1%	24.8%
Lemiale, 2015	52	3.00	3.05	48	2.60	3.80	0.40	[−0.96; 1.76]	3.4%	18.7%
Rittayamai, 2015	20	1.60	1.70	20	3.70	2.40	−2.10	[−3.39; −0.81]	3.7%	19.3%
Vourc'h, 2019	47	3.60	0.70	43	2.60	0.80	1.00	[0.69; 1.31]	63.8%	25.5%
**Common effect model**	**218**			**211**			**0.75**	**[0.50; 1.00]**	**100.0%**	**--**
**Random effects model**							**0.16**	**[−0.96; 1.27]**	**--**	**100.0%**

Heterogeneity: $I^2 = 82\%$, $\tau^2 = 1.2382$, $p < 0.01$

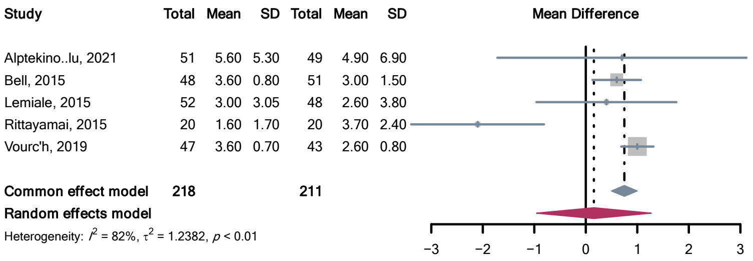

E. Length of ICU stay

Study	HFNC Total	HFNC Mean	HFNC SD	NIV Total	NIV Mean	NIV SD	MD	95%−CI	Weight (common)	Weight (random)
Alptekino..lu, 2021	51	5.69	7.77	49	4.57	3.80	1.12	[−1.26; 3.50]	1.0%	3.4%
Azoulay, 2018	388	8.70	7.44	388	7.75	6.70	0.95	[−0.05; 1.94]	5.5%	11.1%
Coudroy, 2022	154	7.76	6.74	145	8.41	7.49	−0.65	[−2.27; 0.97]	2.1%	6.2%
Grieco, 2021	55	12.83	13.70	54	10.06	9.90	2.77	[−1.71; 7.25]	0.3%	1.1%
Lemiale, 2016	90	9.76	8.29	90	13.63	19.59	−3.87	[−8.27; 0.52]	0.3%	1.1%
Liu, 2018	40	3.49	0.68	40	4.27	0.84	−0.78	[−1.11; −0.45]	48.8%	19.5%
Qiao, 2021	50	13.90	4.80	48	15.50	5.50	−1.60	[−3.65; 0.45]	1.3%	4.4%
Ospina−Tasco..n, 2021	99	8.41	6.02	100	10.76	9.78	−2.35	[−4.61; −0.10]	1.1%	3.7%
Perkins, 2022	416	10.50	15.60	370	9.60	14.10	0.90	[−1.18; 2.98]	1.3%	4.3%
Stephan, 2015	414	6.70	4.46	416	6.70	4.46	0.00	[−0.61; 0.61]	14.8%	16.0%
Vourc'h, 2019	46	3.10	1.60	47	3.30	2.40	−0.20	[−1.03; 0.63]	8.0%	13.1%
Zeng, 2019	62	7.23	1.52	62	7.94	1.83	−0.71	[−1.30; −0.12]	15.6%	16.2%
**Common effect model**	**1865**			**1809**			**−0.50**	**[−0.73; −0.26]**	**100.0%**	**--**
**Random effects model**							**−0.32**	**[−0.80; 0.16]**	**--**	**100.0%**

Heterogeneity: $I^2 = 58\%$, $\tau^2 = 0.2766$, $p < 0.01$

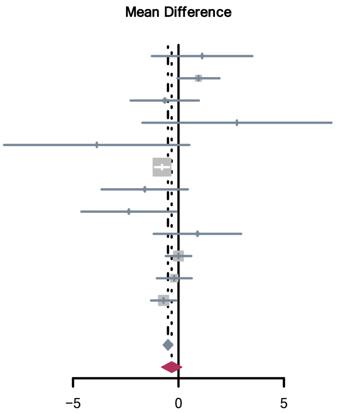

F. Length of hospital stay

Study	HFNC Total	HFNC Mean	HFNC SD	NIV Total	NIV Mean	NIV SD	MD	95%−CI	Weight (common)	Weight (random)
Alptekino..lu, 2021	51	34.32	27.32	49	36.48	28.40	−2.16	[−13.09; 8.77]	0.3%	1.3%
Azoulay, 2018	388	26.10	19.35	388	28.05	20.09	−1.95	[−4.72; 0.83]	5.1%	8.8%
Coudroy, 2022	154	16.46	11.23	145	17.76	14.23	−1.30	[−4.22; 1.62]	4.6%	8.4%
Grieco, 2021	55	26.60	23.60	54	21.71	12.19	4.89	[−2.14; 11.92]	0.8%	2.7%
Lemiale, 2016	90	29.99	27.88	90	34.47	24.86	−4.48	[−12.20; 3.24]	0.7%	2.3%
Liu, 2018	40	18.86	3.95	40	19.19	3.77	−0.33	[−2.02; 1.36]	13.6%	11.8%
Nair, 2021	55	9.71	4.57	54	9.00	4.57	0.71	[−1.01; 2.42]	13.3%	11.8%
Ospina−Tasco..n, 2021	99	13.76	8.28	100	15.41	10.53	−1.65	[−4.28; 0.98]	5.6%	9.1%
Perkins, 2022	416	18.30	20.00	370	17.10	18.00	1.20	[−1.46; 3.86]	5.5%	9.1%
Stephan, 2015	414	14.75	9.67	416	14.35	8.18	0.40	[−0.82; 1.62]	26.3%	13.1%
Wang, 2018	28	13.40	3.50	28	17.50	6.20	−4.10	[−6.74; −1.46]	5.6%	9.1%
Zhao, 2019	80	12.50	4.14	80	16.49	5.15	−3.99	[−5.44; −2.54]	18.6%	12.5%
**Common effect model**	**1870**			**1814**			**−1.00**	**[−1.63; −0.38]**	**100.0%**	**--**
**Random effects model**							**−1.10**	**[−2.38; 0.18]**	**--**	**100.0%**

Heterogeneity: $I^2 = 71\%$, $\tau^2 = 2.8567$, $p < 0.01$

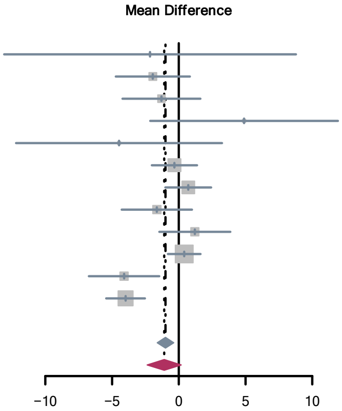

G. 24h-PaO_2/FiO_2

Study	HFNC Total	HFNC Mean	HFNC SD	NIV Total	NIV Mean	NIV SD	MD	95%−CI	Weight (common)	Weight (random)
Andino, 2020	24	129.70	27.30	22	131.70	44.80	−2.00	[−23.67; 19.67]	0.0%	12.1%
Azoulay, 2018	388	176.00	1.70	388	149.10	1.99	26.90	[26.64; 27.16]	98.5%	13.3%
Grieco, 2021	55	133.70	37.80	54	190.80	74.60	−57.10	[−79.36; −34.84]	0.0%	12.0%
Nair, 2021	55	134.70	78.80	54	157.60	82.60	−22.90	[−53.22; 7.42]	0.0%	11.1%
Qiao, 2019	50	287.70	14.70	48	275.50	14.60	12.20	[6.40; 18.00]	0.2%	13.2%
Stephan, 2015	416	261.00	19.30	414	197.60	15.60	63.40	[61.01; 65.79]	1.2%	13.3%
Vourc'h, 2019	47	129.90	54.00	43	106.90	62.60	23.00	[−1.26; 47.26]	0.0%	11.8%
Zhao, 2019	80	175.50	28.54	80	167.49	30.05	8.01	[−1.07; 17.09]	0.1%	13.1%
**Common effect model**	**1115**			**1103**			**27.26**	**[27.01; 27.52]**	**100.0%**	**--**
**Random effects model**							**7.76**	**[−16.94; 32.45]**	**--**	**100.0%**

Heterogeneity: $I^2 = 99\%$, $\tau^2 = 1190.6730$, $p < 0.01$

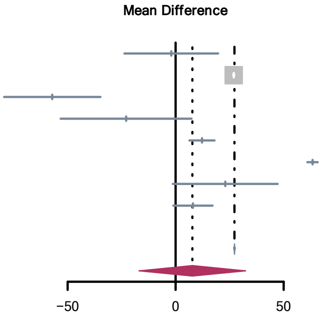

Fig.S4 Network plot of Length of ICU stay(A), Length of hospital stay (B), and 24h-PaO_2/FiO_2 (C)*

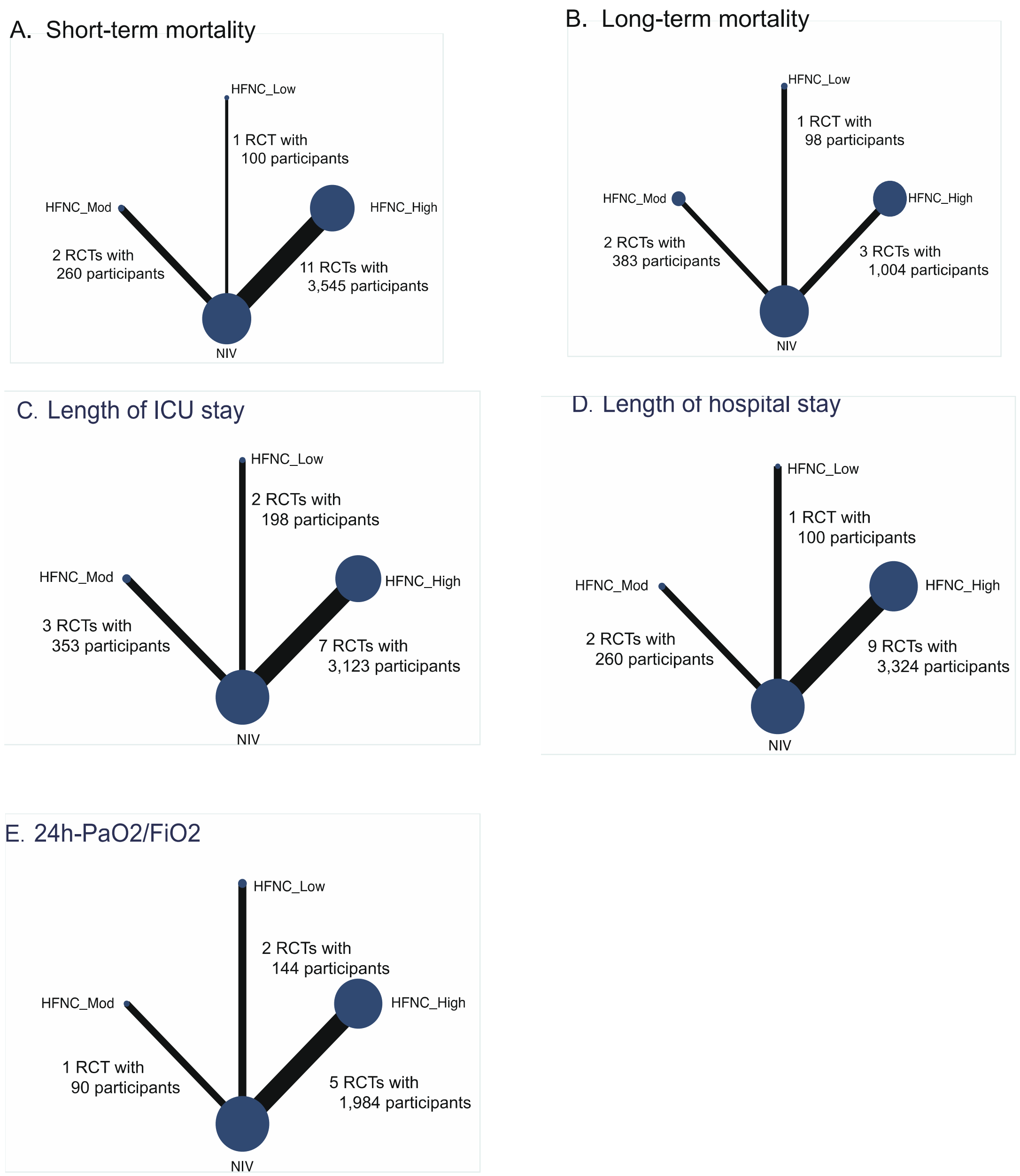

* The size of the node represents the number of participants who received the intervention. The thickness of lines connecting nodes represents the number of trials for that comparison.

Fig.S5 Line chart and bar chart of the surface under the cumulative ranking curve (SUCRA) values*

A. Short-term mortality

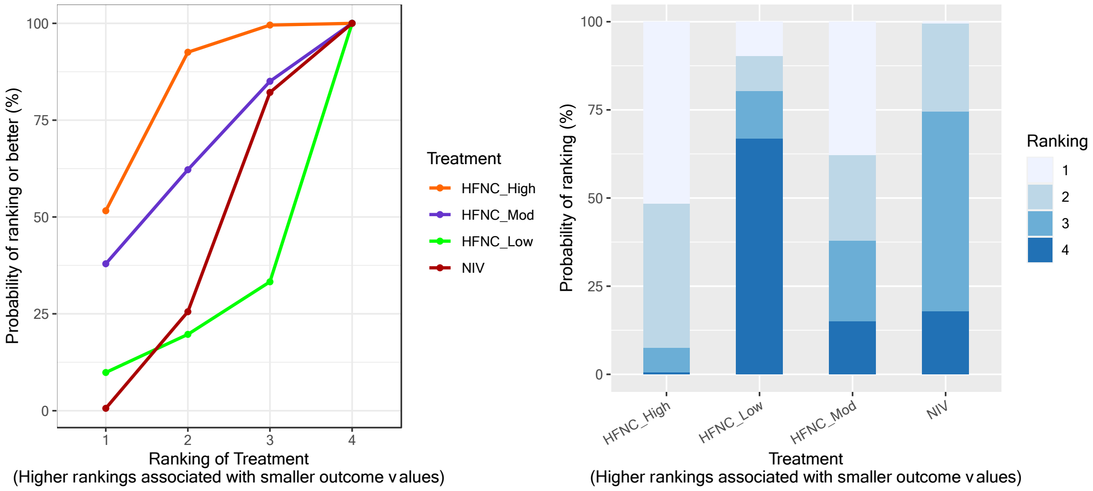

B. Long-term mortality

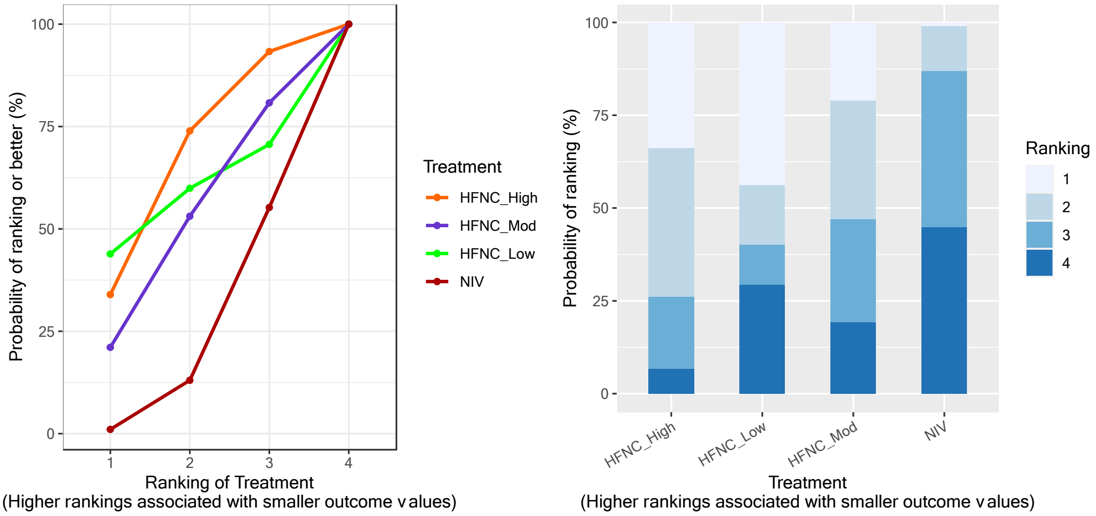

C. Length of ICU stay

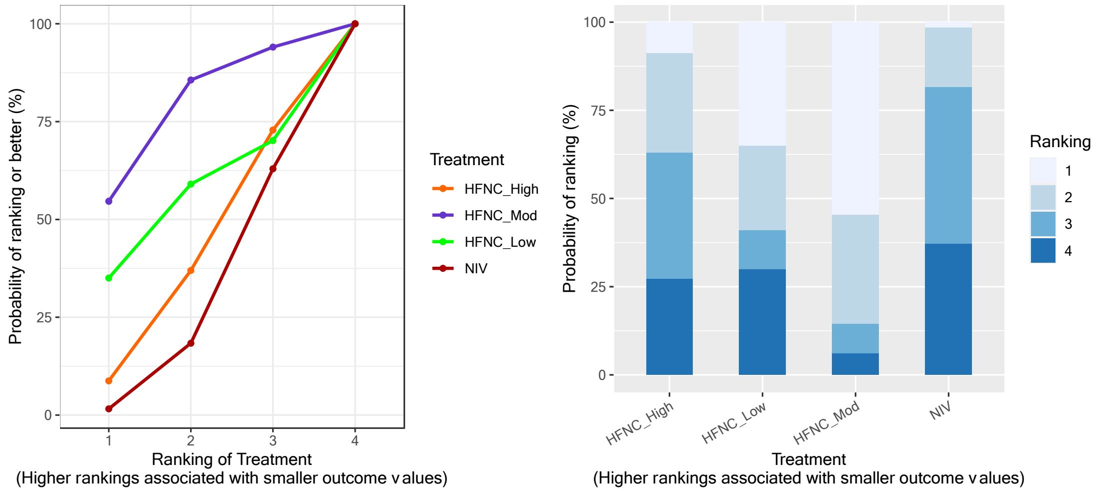

D. Length of hospital stay

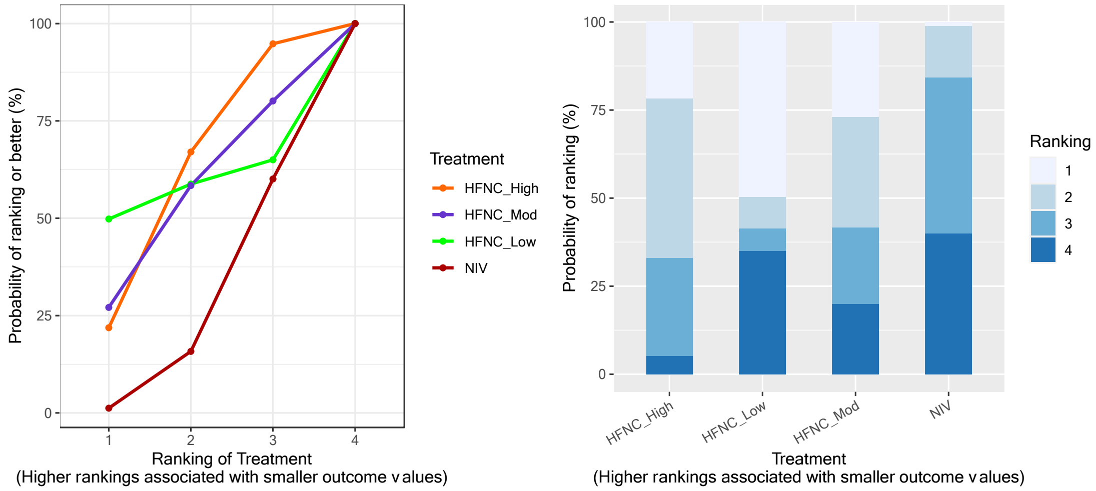

E. 24h-PaO_2/FiO_2

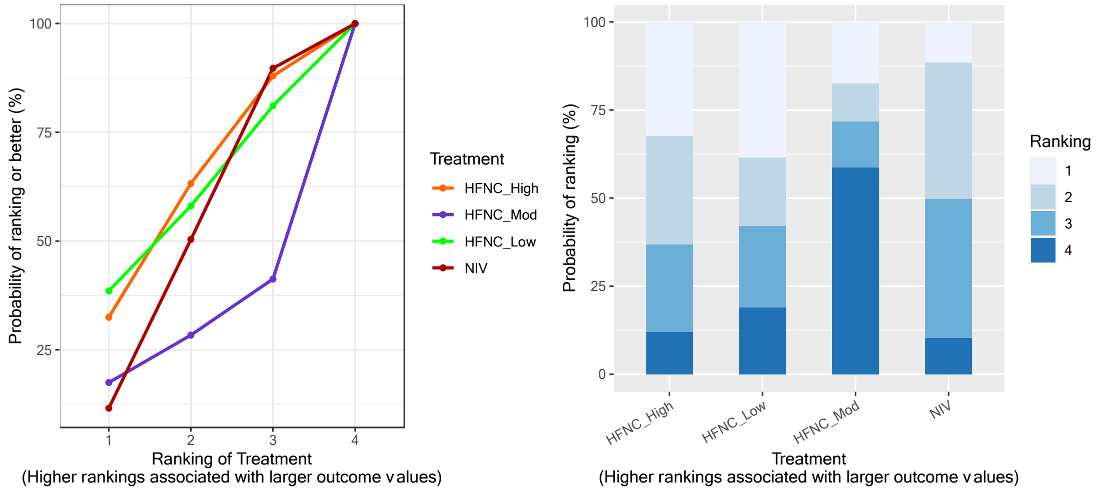

* The x-axis is the ranking of the initial flow rate setting, and the y-axis is the cumulative probability of a particular ranking.
